# Complications and mortality of non-typhoidal salmonella invasive disease: a global systematic review and meta-analysis

**DOI:** 10.1016/S1473-3099(21)00615-0

**Published:** 2022-05

**Authors:** Christian S Marchello, Megan Birkhold, John A Crump, Vacc-iNTS consortium collaborators, Laura B. Martin, Michael O. Ansah, Gianluca Breghi, Rocio Canals, Fabio Fiorino, Melita A. Gordon, Jong-Hoon Kim, Mainga Hamaluba, Brama Hanumunthadu, Jan Jacobs, Samuel Kariuki, Stefano Malvolti, Carsten Mantel, Florian Marks, Donata Medaglini, Vittal Mogasale, Chisomo L. Msefula, Esther Muthumbi, Tonney S. Niyrenda, Robert Onsare, Ellis Owusu-Dabo, Elena Pettini, Maheshi N. Ramasamy, Bassiahi A. Soura, Tiziana Spadafina, Bieke Tack

**Affiliations:** a Centre for International Health, University of Otago, Dunedin, New Zealand; b Department of Surgery, University of Maryland School of Medicine, Baltimore, MD, USA

## Abstract

**Background:**

Non-typhoidal salmonella can cause serious, life-threatening invasive infections involving the bloodstream and other normally sterile sites. We aimed to systematically review the prevalence of complications and case-fatality ratio (CFR) of non-typhoidal salmonella invasive disease to provide contemporary global estimates and inform the development of vaccine and non-vaccine interventions.

**Methods:**

We did a global systematic review and meta-analysis of studies investigating the complications and mortality associated with non-typhoidal salmonella invasive disease. We searched Embase, MEDLINE, Web of Science, and PubMed for peer-reviewed, primary research articles published from database inception up to June 4, 2021, with no restrictions on language, country, date, or participant demographics. Only studies reporting the proportion of complications or deaths associated with non-typhoidal salmonella invasive disease, confirmed by culture of samples taken from a normally sterile site (eg, blood or bone marrow) were included. We excluded case reports, case series, policy reports, commentaries, editorials, and conference abstracts. Data on the prevalence of complications and CFR were abstracted. The primary outcomes were to estimate the prevalence of complications and CFR of non-typhoidal salmonella invasive disease. We calculated an overall pooled CFR estimate and pooled CFR stratified by UN region, subregion, age group, and by serovar when available with a random-effects meta-analysis. A risk-of-bias assessment was done, and heterogeneity was assessed with Cochran's Q Test, *I*^2^, and τ^2^. This study was done in accordance with the Preferred Reporting Items for Systematic Reviews and Meta-Analyses, and is registered with PROSPERO, CRD42020202293.

**Findings:**

The systematic review returned a total of 8770 records. After duplicates were removed, 5837 titles and abstracts were screened, yielding 84 studies from 35 countries after exclusions. Of these included studies, 77 (91·7%) were hospital-based and 66 (78·6%) were located in Africa or Asia. Among 55 studies reporting non-typhoidal salmonella disease-associated complications, a total of 45 different complications were reported and 1824 complication events were identified among 6974 study participants. The most prevalent complication was septicaemia, occurring in 171 (57·2%) of 299 participants, followed by anaemia in 580 (47·3%) of 1225 participants. From 81 studies reporting the CFR of non-typhoidal salmonella invasive disease, the overall pooled CFR estimate was 14·7% (95% CI 12·2–17·3). When stratified by UN region, the pooled CFR was 17·1% (13·6–21·0) in Africa, 14·0% (9·4–19·4) in Asia, 9·9% (6·4–14·0) in Europe, and 9·6% (0·0–25·1) in the Americas. Of all 84 studies, 66 (78·6%) had an overall high risk of bias, 18 (21·4%) had a moderate risk, and none had a low risk. Substantial heterogeneity (*I*^2^>80%) was observed in most (15 [65·2%] of 23) CFR estimates.

**Interpretation:**

Complications were frequent among individuals with non-typhoidal salmonella invasive disease and approximately 15% of patients died. Clinicians, especially in African countries, should be aware of non-typhoidal salmonella invasive disease as a cause of severe febrile illness. Prompt diagnoses and management decisions, including empiric antimicrobial therapy, would improve patient outcomes. Additionally, investments in improving clinical microbiology facilities to identify non-typhoidal salmonella and research efforts towards vaccine development and non-vaccine prevention measures would prevent non-typhoidal salmonella invasive disease-associated illness and death.

**Funding:**

EU Horizon 2020 research and innovation programme.

## Introduction

Non-typhoidal salmonella typically causes acute enterocolitis that is mild and self-limiting in most people.^1^ The illness can also present as a febrile invasive disease often in the absence of diarrhoea, with bacteraemia, meningitis, or focal infections that, if untreated or improperly treated, can be fatal.^2^, ^3^, ^4^ At-risk populations for non-typhoidal salmonella invasive disease include infants and young children, older individuals, immunocompromised or malnourished individuals, and those with recent malaria or HIV infection.^1^, ^2^, ^3^, ^5^, ^6^, ^7^

There were an estimated 535 000 non-typhoidal salmonella invasive disease illnesses and 77 500 deaths due to this disease in 2017.^8^ Of all non-typhoidal salmonella invasive disease illnesses worldwide, most occur in sub-Saharan Africa, where non-typhoidal salmonella is a leading cause of community-onset bloodstream infection.^8^, ^9^, ^10^, ^11^ Most high-income countries have national passive or active surveillance systems in place to monitor the epidemiology of both non-typhoidal salmonella-associated diarrhoea and bloodstream infection, with findings often available in surveillance reports that were outside the scope of our systematic review. By contrast, sentinel surveillance studies are needed to gather such data in low-resource settings. The incidence of non-typhoidal salmonella invasive disease often exceeds 100 illnesses per 100 000 individuals per year in some populations in sub-Saharan Africa, and can exceed 1000 illnesses per 100 000 per year in young children in this region.^12^ Due to clinical similarities with other febrile illnesses, early and accurate diagnosis and treatment of non-typhoidal salmonella invasive disease can be difficult.^13^ Recognised severe complications include sepsis, osteomyelitis, lung infections, and septic arthritis.^1^, ^2^ Previous studies report case-fatality ratios (CFRs) of 20–28% in Mali,^14^, ^15^ Bangladesh,^16^ and Vietnam,^6^ with CFRs as high as 47% in adults with HIV in Malawi.^17^

**Research in context**

**Evidence before this study**

We systematically searched Embase, MEDLINE, Web of Science, and PubMed with no restrictions on language, study location, date, or participant demographics using the search terms non-typhoidal salmonella, mortality, morbidity, died, fatal, and complications. We included peer-reviewed, research articles published from database inception up to May 21, 2020, reporting the complications and mortality of non-typhoidal salmonella invasive disease. Case reports, case-series, policy reports, commentaries, editorials, and conference abstracts were excluded. Available reviews on non-typhoidal salmonella invasive disease-related complications and mortality were scarce. Those identified used geographically restricted search strategies, or focused on specific domains, such as antimicrobial resistance. It was estimated that non-typhoidal salmonella invasive disease caused approximately 535 000 illnesses and 775 00 deaths in 2017. Complications of non-typhoidal salmonella invasive disease, such as focal abscesses, meningitis, osteomyelitis, lung infections, and septic arthritis, can be severe, debilitating, and life-threatening.

**Added value of this study**

In this systematic review and meta-analysis, we update and expand on previous work to present contemporary global estimates of the proportion of patients with non-typhoidal salmonella invasive disease who develop complications, and pooled estimates of case-fatality ratios for culture-confirmed, non-typhoidal salmonella invasive disease globally, by UN region, UN subregion, and stratified by age, when possible. Since January, 2015, an additional 22 eligible studies were published on non-typhoidal salmonella invasive disease mortality, adding substantial new data on this disease that had not been collated previously. Our comprehensive review and analysis is the first to provide contemporary information on the complications and mortality associated with non-typhoidal salmonella invasive disease globally, including in regions without up-to-date, robust national surveillance data.

**Implications of all the available evidence**

Although the predominance of hospital-based studies is likely to have biased our results towards severe non-typhoidal salmonella invasive disease, we identify frequent serious complications and high case fatality ratios that could be averted with prevention measures. Clinicians, especially in African countries, should be aware of non-typhoidal salmonella invasive disease as a cause of severe febrile illness, and consider non-typhoidal salmonella in empiric antimicrobial therapy choices. Investments in clinical microbiology facilities to identify non-typhoidal salmonella and antimicrobial susceptibility profiles would aid in diagnosis and management decisions, with the potential to improve outcomes. Additionally, the development and deployment of an effective and safe bivalent or multivalent non-typhoidal salmonella vaccine has the potential to avert substantial illness and death.

There are more than 2500 recognised serovars of *Salmonella enterica.*^18^ However, two serovars, Typhimurium and Enteritidis, have been identified as the most common causes of non-typhoidal salmonella invasive disease in humans.^10^, ^11^, ^19^ To confirm the diagnosis of non-typhoidal salmonella invasive disease, and to obtain isolates for serotyping and antimicrobial susceptibility testing, the culture of blood, cerebrospinal fluid, bone marrow, or another normally sterile site is required.^1^, ^20^ Unfortunately, microbiology services are often not available in low-resource areas. Additionally, antimicrobial resistance among non-typhoidal salmonella is prevalent in sub-Saharan Africa.^20^, ^21^, ^22^ Multidrug resistance and resistance to third-generation cephalosporins, including ceftriaxone, has emerged among non-typhoidal salmonella that will exacerbate already poor outcomes for patients.^23^ The development and deployment of an efficacious and safe vaccine, which is a major aim of the Vacc-iNTS research project, would be valuable to prevent non-typhoidal salmonella invasive disease, which is becoming increasingly difficult to treat.^24^, ^25^

Previous systematic reviews of non-typhoidal salmonella invasive disease have been limited by geographical location^9^ or restricted to describing antimicrobial resistance among isolates.^23^ Contemporary, global estimates of non-typhoidal salmonella invasive disease morbidity and mortality are needed to inform decisions on future vaccine development and non-vaccine intervention efforts. We therefore aimed to conduct a systematic review and meta-analysis of the prevalence of complications and the CFR in patients with non-typhoidal salmonella invasive disease.

## Methods

### Search strategy and selection criteria

We did a global systematic review and meta-analysis of studies investigating the complications and mortality of non-typhoidal salmonella invasive disease. We did a literature search of Embase, MEDLINE, Web of Science, and PubMed on May 21, 2020, for peer-reviewed, primary research articles published from database inception up to the date of the literature search; an updated search was done on June 4, 2021 (appendix pp 2–7). Key search terms included non-typhoidal salmonella, specific serovars (eg, Typhimurium), mortality, morbidity, died, fatal, and complications. We did not restrict the search by language, country, date, or participant demographics. References of included articles were also screened.

We included studies reporting the proportion of complications or deaths from non-typhoidal salmonella invasive disease, confirmed by culture of samples taken from a normally sterile site (eg, blood or bone marrow). Included studies recruited participants of any age reporting the number of patients with non-typhoidal salmonella invasive disease who had a complication or died. We excluded case reports, case series, policy reports, commentaries, editorials, and conference abstracts. Complications were author-defined. For example, in the study by Shahunja and colleagues,^16^ pneumonia was diagnosed based on the physician's interpretation of clinical findings and the chest radiograph, and clinical sepsis was defined as presence or suspected presence of infection plus any two author-defined characteristics. We did not create a predetermined list of complications to be abstracted. We also did not redefine or standardise complications, for example, by using laboratory thresholds for classifying anaemia or criteria for author-defined sepsis or septicaemia.

Titles and abstracts from each database search result were downloaded and imported into Endnote X8. Duplicates were removed by Endnote X8 and collated into one reference list to be uploaded to the online systematic review tool Rayyan (Qatar Computing Research Institute, Doha, Qatar) for screening.^26^ Screening of titles and abstracts and full-text portable document formats was done in parallel by two authors (CSM and MB), and a third author (JAC) was consulted if conflicts could not be resolved through discussion. Data were then abstracted (by CSM and MB) by use of Google Forms.

This study was done according to the Preferred Reporting Items for Systematic Reviews and Meta-Analyses.^27^ This was a study of published data and, as such, institutional review board approval was not required. The study protocol is available online.

### Data analysis

Study characteristics abstracted included study country and location, UN region and subregion, study setting, study design, data collection start and end date, normally sterile site cultured, and the age group of participants (children aged ≤15 years, adults aged >15 years, or mixed ages; appendix pp 8–20). Age groups were defined on the basis of inclusion criteria or age-range data provided in the results.

After abstracting study characteristics, CSM and MB abstracted data on complications and mortality. As presented in the primary articles by the study authors, we documented the number of participants with non-typhoidal salmonella invasive disease, how many deaths occurred among those with non-typhoidal salmonella invasive disease, and the number and type of complications. When available, we stratified the data by *S enterica* serovar. We also abstracted mortality data among participants with non-typhoidal salmonella invasive disease who were or were not coinfected with HIV and malaria. HIV and malaria coinfection status were author-defined. We did not contact the authors of included studies for individual patient-level data. The final dataset was reviewed by a third author (JAC) for completeness and accuracy.

The primary outcomes were to estimate the prevalence of complications and CFR of non-typhoidal salmonella invasive disease. The prevalence of complications was calculated by dividing the number of complication events that occurred by the number of participants with non-typhoidal salmonella invasive disease. All denominators for complications were calculated using patients for whom data were available. Of note, we could not reliably ascertain from the data provided in eligible studies if a single participant had multiple complications or if multiple participants had different complications. When explicitly described by authors, we stratified extraintestinal focal infections by type (eg, as an abscess or meningitis). When an extraintestinal complication was only described by the authors as a focal infection or similar, we classified the complication as a non-specific extraintestinal focal infection.

We calculated the CFR by dividing the number of deaths by the number of participants with non-typhoidal salmonella invasive disease. We did a meta-analysis in MetaXL version 5.3 (EpiGear International Pty, Sunrise Beach, QLD, Australia) using the DerSimonian-Laird random-effects model with double arcsine transformation to report pooled CFR estimates.^28^, ^29^ Forest plots were generated with natural scales. Heterogeneity was assessed by use of Cochran's Q test, *I*^2^, and τ^2^. We also meta-analysed HIV and malaria mortality data using a random-effects model to find out whether there was an association between the risk of death among participants with non-typhoidal salmonella invasive disease who were or were not coinfected with HIV, and between the risk of death among participants with non-typhoidal salmonella invasive disease who were or were not coinfected with malaria. We calculated pooled odds ratios (ORs) using inverse variance heterogeneity,^30^ and ORs were considered significant if the 95% CI did not cross 1·0.

Risk of bias was assessed according to two main domains, guided by prevalence, incidence, and non-randomised study bias assessment tools.^31^, ^32^, ^33^ For the selection and recruitment domain, we evaluated study design, study setting, and patient selection. In the measurement and reporting domain, we evaluated definitions for complications, patient follow-up, and microbiology methods. We scored each question as unknown, low, or high risk, and assigned each study as having a low, moderate, or high risk of bias (appendix pp 21–22).

The protocol was submitted to PROSPERO on Aug 2, 2020, and registered on Sept 1, 2020 (CRD42020202293).

### Role of the funding source

The funder of the study had no role in study design, data collection, data analysis, data interpretation, or writing of the report.

## Results

**Figure 1:**
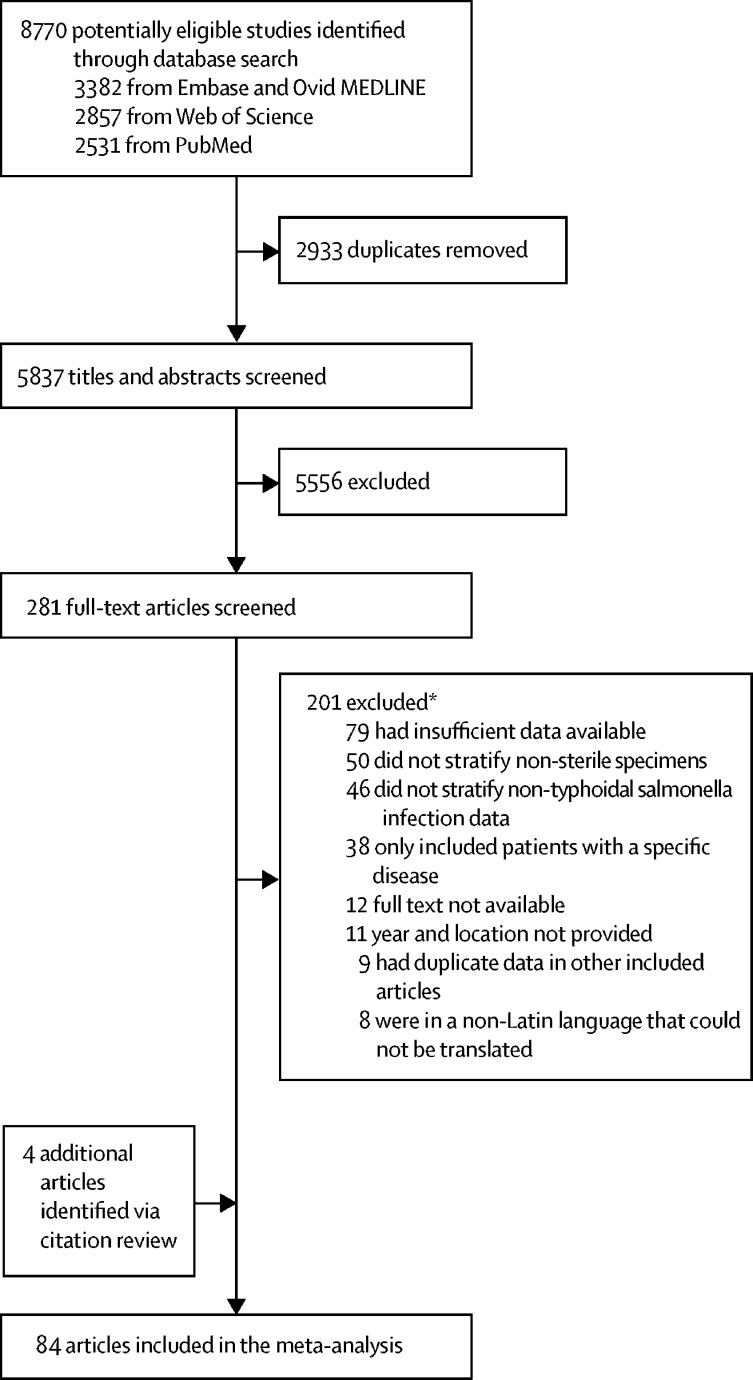
Study selection for a systematic review and meta-analysis of complications and mortality of non-typhoidal salmonella invasive disease worldwide from 1971 up to 2019 *The sum of articles excluded for each reason exceeds the total number of studies excluded because a study could be excluded for more than one reason.

Our search strategy returned 8770 records. We screened 5837 titles and abstracts for inclusion after 2933 duplicates were removed. Of 5837 titles and abstracts, 281 (4·8%) proceeded for full-text review. We excluded 201 (71·5%) studies, resulting in 80 eligible studies for analysis. When screening the citations of the 80 studies, we identified an additional four studies that met our inclusion criteria, yielding a total of 84 eligible studies for analysis (figure 1).^6^, ^15^, ^16^, ^34^, ^35^, ^36^, ^37^, ^38^, ^39^, ^40^, ^41^, ^42^, ^43^, ^44^, ^45^, ^46^, ^47^, ^48^, ^49^, ^50^, ^51^, ^52^, ^53^, ^54^, ^55^, ^56^, ^57^, ^58^, ^59^, ^60^, ^61^, ^62^, ^63^, ^64^, ^65^, ^66^, ^67^, ^68^, ^69^, ^70^, ^71^, ^72^, ^73^, ^74^, ^75^, ^76^, ^77^, ^78^, ^79^, ^80^, ^81^, ^82^, ^83^, ^84^, ^85^, ^86^, ^87^, ^88^, ^89^, ^90^, ^91^, ^92^, ^93^, ^94^, ^95^, ^96^, ^97^, ^98^, ^99^, ^100^, ^101^, ^102^, ^103^, ^104^, ^105^, ^106^, ^107^, ^108^, ^109^, ^110^, ^111^, ^112^, ^113^, ^114^

**Figure 2:**
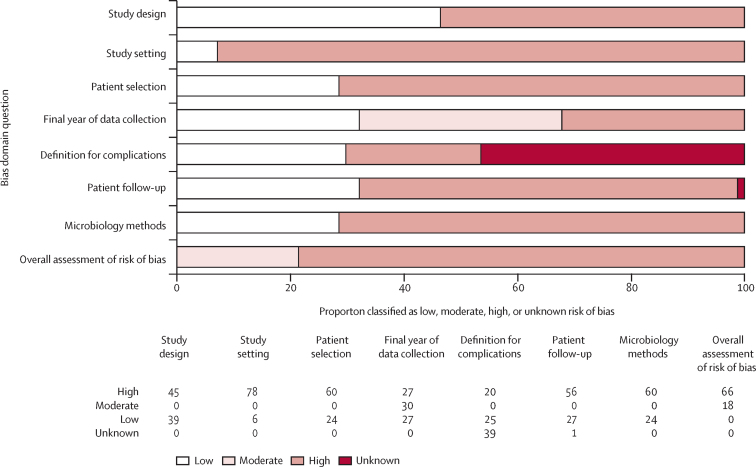
Quality assessment for the risk of bias of included studies on the complications and mortality associated with non-typhoidal salmonella invasive disease worldwide from 1971 up to 2019

Among these 84 studies, data were collected between 1971 and 2019 in four of the five UN regions: 33 (39·3%) in Africa, 33 (39·3%) in Asia, 13 (15·5%) in Europe, five (6·0%) in the Americas, and none in Oceania (appendix pp 26–29). Regarding the UN subregions, 19 (22·6%) studies collected data in eastern Africa, followed by 14 (16·7%) in eastern Asia, and nine (10·7%) in southeastern Asia. Ten UN subregions had one to seven studies, whereas nine subregions had no studies (appendix pp 26–29). Among the 84 studies, 55 (65·5%) had available data on non-typhoidal salmonella invasive disease-related complications and 81 (96·4%) reported CFRs; three studies^61^, ^83^, ^102^ had data on complications but did not report CFRs. A total of 39 (46·4%) studies recruited children only, 26 (31·0%) recruited both children and adults, and 19 (22·6%) recruited adults only. 77 (91·7%) studies recruited patients from a hospital setting, six (7·1%) were not hospital-based, and one (1·2%) recruited patients from both hospital and outpatient settings.^15^ 72 (85·7%) studies reported isolating non-typhoidal salmonella from blood cultures only, three (3·6%) from either blood or cerebrospinal fluid cultures, and the remaining nine (10·7%) from cultures of blood, cerebrospinal fluid, or another normally sterile fluid, such as pleural fluid or synovial fluid. Of all 84 studies, 66 (78·6%) had an overall high risk of bias, 18 (21·4%) had a moderate risk, and none had a low risk (figure 2; appendix pp 23–25).

Among the 55 studies reporting complications of non-typhoidal salmonella invasive disease, confirmed by cultures of a normally sterile site, 45 different complications were reported, and 1824 complication events were identified among 6974 study participants (table 1). The most prevalent complication was septicaemia, occurring in 171 (57·2%) of 299 participants, followed by anaemia in 580 (47·3%) of 1225 participants. The most commonly reported complication was an abscess, with 19 estimates reporting this occurring in 47 (2·9%) of 1609 participants, followed by 17 estimates reporting recurrence in 110 (4·8%) of 2229 participants, and 16 estimates reporting pneumonia in 232 (14·4%) of 1619 participants. Among severe, life-threatening complications, encephalopathy was reported in eight (20·0%) of 40 participants, extraintestinal focal infections in 66 (9·2%) of 721, pleuropulmonary infection in 17 (8·3%) of 204, mycotic aneurysm in 124 (6·2%) of 1991, encephalitis in three (4·8%) of 62, and endocarditis in six (1·6%) of 366.

**Table 1:** Complications of non-typhoidal salmonella invasive disease from 1971 up to 2019 globally by age group

	**Adults aged >15 years**	**Children aged ≤15 years**	**Mixed ages**	**Total**	**Number of estimates**
Any complication	426/5155 (8·3%)	1060/7926 (13·4%)	338/5847 (5·8%)	1824/6974 (26·2%)	188
Septicaemia	ND	167/273 (61·2%)	4/26 (15·4%)	171/299 (57·2%)	2
Anaemia	ND	537/1156 (46·5%)	43/69 (62·3%)	580/1225 (47·3%)	12
Acute kidney injury	ND	ND	10/40 (25·0%)	10/40 (25·0%)	1
Encephalopathy	ND	ND	8/40 (20·0%)	8/40 (20·0%)	1
Shock	105/515 (20·4%)	2/45 (4·4%)	ND	107/560 (19·1%)	6
Pneumonia	13/129 (10·1%)	193/1209 (16·0%)	26/281 (9·3%)	232/1619 (14·3%)	16
Ventriculitis	ND	1/9 (11·1%)	ND	1/9 (11·1%)	1
Septic shock	23/206 (11·2%)	2/20 (10·0%)	11/105 (10·5%)	36/331 (10·9%)	6
Disseminated intravascular coagulation	ND	1/10 (10·0%)	ND	1/10 (10·0%)	1
Lung infection	ND	1/10 (10·0%)	ND	1/10 (10·0%)	1
Nephropathy	ND	ND	4/40 (10·0%)	4/40 (10·0%)	1
Seizures	ND	22/216 (10·2%)	3/40 (7·5%)	25/256 (9·8%)	4
Jaundice	ND	13/134 (9·7%)	ND	13/134 (9·7%)	3
Non-specific extraintestinal focal infections	52/200 (26·0%)	2/246 (0·8%)	12/275 (4·4%)	66/721 (9·2%)	5
Convulsions	ND	10/110 (9·1%)	1/20 (5·0%)	11/130 (8·5%)	3
Haemorrhage	ND	ND	5/60 (8·3%)	5/60 (8·3%)	2
Pleuropulmonary infection	17/204 (8·3%)	ND	ND	17/204 (8·3%)	1
Coma	13/79 (16·5%)	3/120 (2·5%)	ND	16/199 (8·0%)	3
Impaired consciousness	ND	3/45 (6·7%)	ND	3/45 (6·7%)	1
Joint swelling	ND	1/15 (6·7%)	ND	1/15 (6·7%)	1
Vascular infection	13/206 (6·3%)	ND	ND	13/206 (6·3%)	1
Mycotic aneurysm	63/564 (11·2%)	ND	61/1427 (4·3%)	124/1991 (6·2%)	7
Urinary tract infection	ND	8/134 (6·0%)	ND	8/134 (6·0%)	2
Altered consciousness	ND	2/36 (5·6%)	ND	2/36 (5·6%)	1
Pancreatitis	ND	ND	2/40 (5·0%)	2/40 (5·0%)	1
Recurrence	61/574 (10·6%)	13/532 (2·4%)	36/1193 (3·0%)	110/2299 (4·8%)	17
Encephalitis	ND	ND	3/62 (4·8%)	3/62 (4·8%)	1
Gangrene of lower limb	ND	2/53 (3·8%)	ND	2/53 (3·8%)	1
Abscess	27/667 (4·0%)	14/568 (2·5%)	6/374 (1·6%)	47/1609 (2·9%)	19
Septic arthritis	2/129 (1·6%)	11/328 (3·4%)	3/109 (2·8%)	16/566 (2·8%)	7
Arthritis	7/159 (4·4%)	10/453 (2·2%)	1/40 (2·5%)	18/652 (2·8%)	7
Osteomyelitis	20/564 (3·5%)	12/624 (1·9%)	6/310 (1·9%)	38/1498 (2·5%)	15
Colitis	ND	ND	1/40 (2·5%)	1/40 (2·5%)	1
Pericarditis	ND	ND	1/40 (2·5%)	1/40 (2·5%)	1
Peritonitis	3/129 (2·3%)	ND	ND	3/129 (2·3%)	1
Myocarditis	ND	ND	1/45 (2·2%)	1/45 (2·2%)	1
Endocarditis	3/210 (1·4%)	ND	3/156 (1·9%)	6/366 (1·6%)	4
Subdural effusion	ND	2/127 (1·6%)	ND	2/127 (1·6%)	2
Colonic perforation	ND	ND	1/64 (1·6%)	1/64 (1·6%)	1
Haemolytic anaemia	ND	1/74 (1·4%)	ND	1/74 (1·4%)	1
Catheter-related infection	1/129 (0·8%)	ND	ND	1/129 (0·8%)	1
Empyema	1/206 (0·5%)	ND	ND	1/206 (0·5%)	1
Necrotising fasciitis	1/206 (0·5%)	ND	ND	1/206 (0·5%)	1

Data are n/N (%), unless otherwise indicated. ND=No data available.

**Figure 3:**
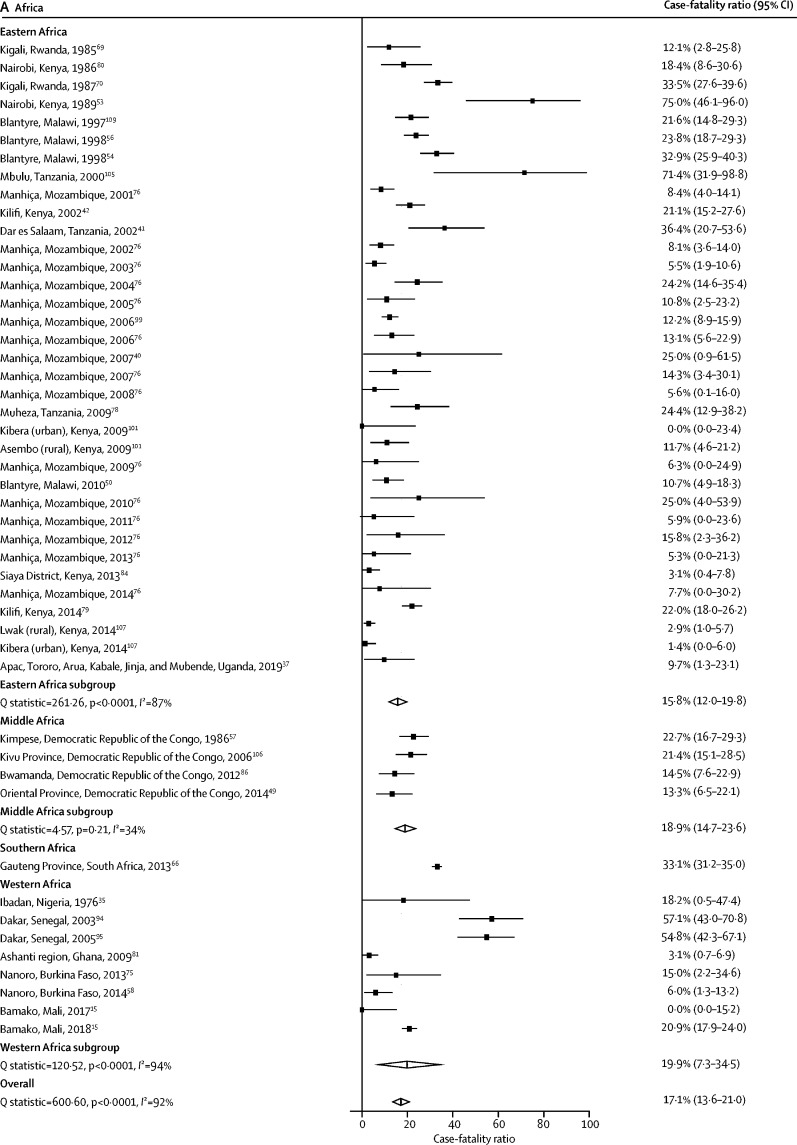

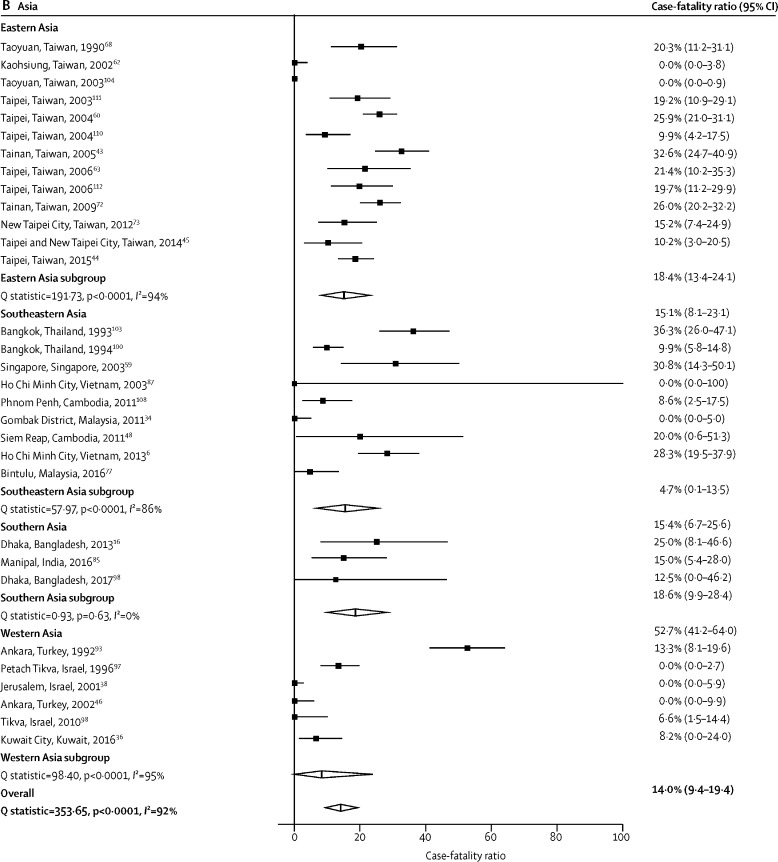
Forest plot of non-typhoidal salmonella case-fatality ratios among study sites in Africa from 1976 up to 2019, by UN subregion (A), and in Asia from 1990 up to 2017, by UN subregion (B)

Of the 81 studies reporting a CFR for non-typhoidal salmonella invasive disease, two (2·5%) included data from multiple study sites,^101^, ^107^ one (1·2%) was done over multiple consecutive years,^77^ and one (1·2%) included both hospital and outpatient settings, 15 resulting in 97 individual CFR estimates. The median CFR across all 97 estimates was 13·3% (IQR 5·5–22·7). The overall pooled CFR estimate was 14·7% (95% CI 12·2–17·3). When stratified by UN region, the pooled CFR was 17·1% (13·6–21·0) in Africa, 14·0% (9·4–19·4) in Asia, 9·9% (6·4–14·0) in Europe, and 9·6% (0·0–25·1) in the Americas (table 2). In Africa, when stratified by UN subregion, the pooled CFR was 33·1% (31·2–35·0) in southern Africa, 19·9% (7·3–34·5) in western Africa, 18·9% (14·7–23·6) in middle Africa, and 15·8% (12·0–19·8) in eastern Africa (table 2; figure 3A). The pooled CFR in adults was 21·0% (16·6–25·7) compared with 12·0% (9·0–15·4) in children (table 2). Of all 97 estimates, 89 (91·8%) were from hospital-based studies, with a pooled CFR of 15·1% (12·5–17·8), and eight (8·2%) were from non-hospital-based studies, with a pooled CFR of 9·9% (2·8–20·0). Substantial heterogeneity (*I*^2^>80%), was observed in 15 (65·2%) of 23 of the pooled CFR estimates (table 2).

**Table 2:** Pooled case-fatality ratio of non-typhoidal salmonella infection from 1971 up to 2019 by UN subregion, age group, serovar, and setting

			**Pooled case-fatality ratio (95% CI)**	**Q statistic**	**I^2^ (95% CI)**	**τ^2^**	CFR estimates*
Global			14·7% (12·2–17·3)	1192·6	92·0 (90·7–93·0)	0·102	97
UN subregion							
	Africa		17·1% (13·6–21·0)	600·6	92·2 (90·4–93·6)	0·093	48
		Eastern Africa	15·8% (12·0–19·8)	261·3	87·0 (82·9–90·1)	0·078	35
		Middle Africa	18·9% (14·7–23·6)	4·6	34·3 (0·0–76·9)	0·004	4
		Southern Africa	33·1% (31·2–35·0)	0	NR	0·000	1
		Western Africa	19·9% (7·3–34·5)	120·5	94·2 (90·7–96·4)	0·204	8
	Americas†		9·6% (0·0–25·1)	21·2	81·1 (56·1–91·9)	0·196	5
	Asia		14·0% (9·4–19·4)	353·6	91·5 (89·0–93·4)	0·137	31
		Eastern Asia	15·1% (8·1–23·1)	191·7	93·7 (91·0–95·7)	0·132	13
		Southeastern Asia	15·4% (6·7–25·6)	58	86·2 (75·8–92·1)	0·119	9
		Southern Asia	18·6% (9·9–28·4)	0·9	0·0 (0·0–96·3)	0·000	3
		Western Asia	8·2% (0·0–24·0)	98·4	94·9 (91·4–97·0)	0·315	6
	Europe		9·9% (6·4–14·0)	37·7	68·2 (43·4–82·1)	0·028	13
		Eastern Europe	15·4% (3·6–32·2)	0	NR	0·000	1
		Northern Europe	15·2% (10·3–20·9)	12·2	75·5 (32·2–91·1)	0·157	4
		Southern Europe	3·4% (0·9–7·2)	3·1	0·0 (0·0–74·9)	0·000	6
		Western Europe	8·8% (0·0–36·9)	6·8	85·2 (40·1–96·4)	0·238	2
Age group							
	Adults aged >15 years		21·0% (16·6–25·7)	67·5	74·8 (60·1–84·1)	0·037	18
	Children aged ≤15 years		12·0% (9·0–15·4)	470·0	89·1 (86·6–91·2)	0·102	52
	Mixed ages		15·4% (10·6–20·9)	547·9	95·3 (94·0–96·2)	0·121	27
*Salmonella enterica* serovar							
	Typhimurium		21·6% (15·6–28·2)	174·3	86·8 (81·6–90·5)	0·087	24
	Enteritidis		15·0% (10·9–19·7)	88·5	71·7 (58·1–80·9)	0·052	26
	Dublin		19·1% (15·0–23·6)	3·2	0·0 (0·0–75·9)	0·000	6
Setting							
	Hospital		15·1% (12·5–17·8)	776·5	88·7 (86·7–90·4)	0·098	89
	Non-hospital		9·9% (2·8–20·0)	374·7	98·1 (97·4–98·7)	0·145	8

NR=not reportable. *I*^2^ values were NR because only one study was available.

* Number of estimates contributing to the pooled estimate.

† Includes North America only.

One study provided CFR data over multiple consecutive years in one location.^76^ In Manhiça, Mozambique, ten (8·4%) of 199 participants died in 2001. The highest CFR was observed in 2010, when three (25·0%) of 12 participants died, and the lowest was in 2013 when one (5·2%) of 19 died (appendix p 32).

Among 12 977 participants with non-typhoidal salmonella invasive disease, 5934 (45·7%) *S enterica* isolates were serovar Typhimurium, 4122 (31·8%) were serovar Enteritidis, 403 (3·1%) were serovar Dublin, 186 (1·4%) were serovar Virchow, 172 (1·3%) were serovar Isangi, and 134 (1·0%) were serovar Choleraesuis (appendix pp 30–31). All other serovars each accounted for less than 1·0% of non-typhoidal salmonella invasive isolates identified. The pooled CFR among *S enterica* serovar Typhimurium infections was 21·6% (15·6–28·2), for serovar Dublin was 19·1% (15·0–23·6), and for serovar Enteritidis was 15·0% (10·9–19·7; table 2). There were insufficient data available to examine CFR associations for other serovars.

Eight studies reported CFRs among participants with non-typhoidal salmonella invasive disease who had HIV coinfection versus those without HIV coinfection.^42^, ^48^, ^66^, ^67^, ^72^, ^84^, ^108^, ^111^ Among 1907 participants with non-typhoidal salmonella invasive disease and HIV coinfection, 595 (31·2%) had died, whereas, among 762 participants with non-typhoidal salmonella invasive disease who did not have HIV coinfection, 128 (16·8%) had died (χ^2^=56·5; p<0·0001). A significantly higher risk of death was observed in participants with HIV coinfection versus those without HIV coinfection, with a pooled OR of 2·4 (95% CI 1·4–4·1; appendix p 33). Four studies reported CFRs in participants with non-typhoidal salmonella invasive disease who had or did not have malaria coinfection, all of whom were children.^37^, ^42^, ^56^, ^105^ Among 138 children with non-typhoidal salmonella invasive disease and malaria coinfection, 24 (17·4%) had died, whereas, among 299 children with non-typhoidal salmonella invasive disease who did not have malaria coinfection, 73 (24·4%) had died (χ^2^=2·3; p=0·13). A non-significantly lower risk of death was observed in participants with malaria coinfection versus those without malaria coinfection, with a pooled OR of 0·6 (95% CI 0·3–1·4; appendix p 34).

## Discussion

Our systematic review and meta-analysis showed that a substantial proportion of patients with non-typhoidal salmonella invasive disease have complications, many of which are life-threatening, including encephalopathy, pleuropulmonary infection, mycotic aneurysm, encephalitis, focal abscesses, and endocarditis. We also estimated that approximately 15% of patients with non-typhoidal salmonella invasive disease die. Despite no restrictions in our search strategy by location, there were no data available from the UN region Oceania, and CFR estimates from UN subregions were only reported in four studies from middle Africa, two studies from southern Africa, and one study from southern Asia. More than half of eligible CFR estimates were from three UN subregions, two of which were in Asia. The scarcity of data from countries and subregions in Africa, which are likely to have a high incidence of this disease, poses an important barrier to fully understanding the epidemiology of non-typhoidal salmonella invasive disease in Africa.

The significantly higher CFR observed in adults than in children in our study could be attributed to the disproportionate role of HIV infection as a major predisposing condition for non-typhoidal salmonella invasive disease in adults.^2^ Although HIV infection is a key driver of non-typhoidal salmonella invasive disease risk in adults, recent malaria infection is an important risk factor for non-typhoidal salmonella invasive disease in children. Unlike HIV, which could be associated with an ongoing risk for excess mortality, malaria itself might often no longer pose a substantial risk of death by the time the child presents with non-typhoidal salmonella invasive disease.^115^

We found a high prevalence of serious complications among patients with non-typhoidal salmonella invasive disease, including acute kidney injury, encephalopathy, pneumonia, haemorrhage, and mycotic aneurysm. Other less prevalent, but serious, life-threatening complications identified included pancreatitis, encephalitis, meningitis, myocarditis, and colonic perforation. Although data on the duration of non-typhoidal salmonella invasive disease-associated complications are scarce, many complications are sufficiently severe to warrant high disability weights.^116^ The impact of the high CFR and occurrence of serious complications is illustrated by comparing the burden of typhoid fever with that of non-typhoidal salmonella invasive disease. Despite non-typhoidal salmonella invasive disease being estimated to cause just 4·9% of the number of illnesses as typhoid fever in 2017, 4·26 million disability-adjusted life-years were attributed to non-typhoidal salmonella invasive disease, more than half as many as typhoid fever.^8^, ^117^ Strengthening burden of disease estimates for non-typhoidal salmonella invasive disease-related complications, for instance, by recording illness duration and the use of appropriate or specific disability weights, would improve awareness and facilitate more accurate comparisons of non-typhoidal salmonella invasive disease with other diseases. The use of more accurate disability-adjusted life-year estimates would support investment decisions in vaccine and non-vaccine prevention strategies, and allow improved targeting of interventions.

Although septicaemia and anaemia were the most prevalent complications reported, we were unable to reinterpret author definitions for complications. According to systemic inflammatory response syndrome criteria, septicaemia is strictly defined as bacteraemia with features of sepsis. We cannot exclude the possibility that some authors might have used a less stringent definition of septicaemia (ie, using septicaemia interchangeably with bacteraemia), resulting in misclassification of sepsis. Some complication categories, such as shock, which is a component of some definitions of sepsis, are unlikely to be mutually exclusive; therefore, the prevalence of these complications might have been overestimated. Additionally, anaemia is a major sequela of HIV and malaria infection, but it is also a recognised host risk factor for non-typhoidal salmonella invasive disease. Consequently, anaemia might be over-reported as a complication of non-typhoidal salmonella invasive disease in our study, since we were unable to ascertain whether it was present before the onset of non-typhoidal salmonella invasive disease. As a result, it was difficult to be confident that author-reported complications met the widely accepted definitions of clinical conditions, and to ascertain whether some clinical observations were actual sequela of non-typhoidal salmonella invasive disease or of pre-existing conditions only.

We showed that non-typhoidal salmonella invasive disease was deadly, with a pooled CFR estimate of 14·7%. The CFR of non-typhoidal salmonella invasive disease is markedly higher than that estimated for other major febrile illnesses, such as malaria, with an estimated CFR of 0·2%,^118^ and typhoid fever, with an estimated CFR of 2·0%.^119^ Although most data in our analysis were from hospital-based studies that could potentially bias our results towards severe disease, we stratified the analysis by setting and observed a non-significant trend for higher CFRs in hospital-based studies than in non-hospital-based studies. Alternatively, active, community-based studies could bias results towards a lower prevalence of complications than hospital-based studies and CFRs as a result of active case-finding, and enhanced diagnosis and treatment. Delays in accessing care leading to later diagnosis and longer time to receive appropriate antimicrobial treatment have been associated with increased CFR among patients with typhoid fever.^119^ Further research investigating whether a similar association is observed among patients with non-typhoidal salmonella invasive disease is warranted.

*S enterica* serovars Typhimurium and Enteritidis were the most prevalent serovars identified in our analysis. Others have reported that most salmonella isolates from invasive infections in sub-Saharan Africa are *S enterica* serovar Typhimurium sequence type 313,^120^ which is predominately multidrug resistant (ie, to ampicillin, chloramphenicol, and co-trimoxazole [trimethoprim-sulfamethoxazole]), and has also shown extensive drug resistance.^121^ Additionally, a high frequency of multidrug resistance has been observed among *S enterica* serovar Enteritidis isolates in Africa.^122^, ^123^ Antimicrobial resistance in the two most prevalent non-typhoidal salmonella serovars will probably lead to poorer outcomes in patients infected with these serovars, making control and prevention efforts more difficult. Non-typhoidal salmonella vaccine development represents an important avenue to prevent complications and deaths from this disease, which is becoming increasingly difficult to treat due to antimicrobial resistance. Vaccines in preclinical or clinical development include live attenuated *S enterica* serovar Typhimurium, non-typhoidal *S enterica* core and O-polysaccharide glycoconjugates, multiple antigen presenting system complexes, and generalised modules for membrane antigens (GMMA) of *S enterica* serovars Enteritidis and Typhimurium expressed on outer membrane vesicles. Phase 1 studies of GMMA vaccines are underway.^25^ Ideally, vaccines to prevent non-typhoidal salmonella invasive disease will need to protect individuals from early in life^12^ and patients with host risk factors for disease, including HIV, malaria, and malnutrition. Our study supports non-typhoidal salmonella vaccine development, especially vaccines focused on *S enterica* serovars Typhimurium and Enteritidis.

Our study had several limitations. Eligible studies were predominantly from Africa and Asia, with some other regions poorly represented. Although there is no test for publication bias in prevalence studies, it is possible that studies identifying a high frequency of complications and high CFRs were more likely to be published than those finding no complications and low CFRs. However, our systematic review identified a large number of studies, and those that were eligible tended to be studies of bloodstream infection prevalence and outcomes. Consequently, it is improbable that outcomes for a specific pathogen would have motivated publication. Eligible publications rarely provided details on modalities for diagnosing comorbidities and case definitions for complications. This lack of detail might have resulted in misclassification of comorbidities and complications and was formally assessed in our bias assessment. We were unable to examine the association between non-typhoidal salmonella invasive disease and comorbidities other than HIV and malaria due to scarce reporting of other comorbidities in eligible studies. We suggest that future studies describing non-typhoidal salmonella invasive disease-related complications provide detailed case definitions. It would also be helpful for the field if such definitions were standardised. Since most eligible studies were hospital-based, it is possible that our findings were biased towards more severe forms of non-typhoidal salmonella invasive disease. However, we found that pooled CFR identified in non-hospital-based studies was not significantly different to that for hospital-based studies. We observed substantial heterogeneity in the meta-analysis, in which the overall global pooled CFR estimate had an *I*^2^ of 92% and some estimates had wide 95% CIs. Although the Cochran's Q test is often underpowered to detect heterogeneity, especially in meta-analyses with less than five included studies or where one study comprises more than 90% of the data.^124^ Many of the meta-analyses in our review consisted of more than 15 studies, and we assessed heterogeneity using several other measures. We attribute most of the heterogeneity to the varying time periods, study locations, and study populations. This degree of heterogeneity was largely expected before our analysis, as we placed no restrictions on time, location, or population in our search strategy to provide as comprehensive review as possible.

In conclusion, we showed that a considerable proportion of patients with non-typhoidal salmonella invasive disease have related complications and death. Prompt diagnosis and management, including consideration of non-typhoidal salmonella invasive disease in the empirical management of severe febrile illness and sepsis in endemic areas, is warranted to improve clinical outcomes. The findings of our systematic review are available to further refine burden of disease estimates for non-typhoidal salmonella invasive disease, the results of which inform investments in the development and deployment of vaccine and non-vaccine interventions to prevent non-typhoidal salmonella invasive disease. Standardisation of definitions and reporting of complications would reduce the risk of bias of future reviews. Additional high-quality research relevant to non-typhoidal salmonella invasive disease burden, including CFR, duration of illness and complications, disability weights, the effect of antimicrobial resistance on outcomes, and host risk factors, especially in regions with scarce data, are needed to better inform investments and decisions on vaccine development and deployment, as well as non-vaccine interventions.

## Data sharing

The protocol for the systematic review is available at: https://www.crd.york.ac.uk/prospero/display_record.php?ID=CRD42020202293. Detailed methods, results, and additional data are available in the manuscript and the associated appendix. Additional data collected from this study can be made available to others with publication. Proposals should be submitted for consideration by the study team via the corresponding author.

## Declaration of interests

We declare no competing interests.

## References

[1] Crump JA, Sjölund-Karlsson M, Gordon MA, Parry CM (2015). Epidemiology, clinical presentation, laboratory diagnosis, antimicrobial resistance, and antimicrobial management of invasive salmonella infections. Clin Microbiol Rev.

[2] Feasey NA, Dougan G, Kingsley RA, Heyderman RS, Gordon MA (2012). Invasive non-typhoidal salmonella disease: an emerging and neglected tropical disease in Africa. Lancet.

[3] Haselbeck AH, Panzner U, Im J, Baker S, Meyer CG, Marks F (2017). Current perspectives on invasive nontyphoidal salmonella disease. Curr Opin Infect Dis.

[4] Roth GA, Abate D, Abate KH (2018). Global, regional, and national age-sex-specific mortality for 282 causes of death in 195 countries and territories, 1980–2017: a systematic analysis for the Global Burden of Disease Study 2017. Lancet.

[5] Graham SM (2010). Nontyphoidal salmonellosis in Africa. Curr Opin Infect Dis.

[6] Phu Huong Lan N, Le Thi Phuong T, Nguyen Huu H (2016). Invasive non-typhoidal salmonella infections in Asia: clinical observations, disease outcome and dominant serovars from an infectious disease hospital in Vietnam. PLoS Negl Trop Dis.

[7] Deen J, von Seidlein L, Andersen F, Elle N, White NJ, Lubell Y (2012). Community-acquired bacterial bloodstream infections in developing countries in south and southeast Asia: a systematic review. Lancet Infect Dis.

[8] GBD 2017 Non-Typhoidal Salmonella Invasive Disease Collaborators (2019). The global burden of non-typhoidal salmonella invasive disease: a systematic analysis for the Global Burden of Disease Study 2017. Lancet Infect Dis.

[9] Uche IV, MacLennan CA, Saul A (2017). A systematic review of the incidence, risk factors and case fatality rates of invasive nontyphoidal salmonella (iNTS) disease in Africa (1966 to 2014). PLoS Negl Trop Dis.

[10] Reddy EA, Shaw AV, Crump JA (2010). Community-acquired bloodstream infections in Africa: a systematic review and meta-analysis. Lancet Infect Dis.

[11] Marchello CS, Dale AP, Pisharody S, Rubach MP, Crump JA (2019). A systematic review and meta-analysis of the prevalence of community-onset bloodstream infections among hospitalized patients in Africa and Asia. Antimicrob Agents Chemother.

[12] Marchello CS, Fiorino F, Pettini E, Crump JA, Vacc-iNTS Consortium Collaborators (2021). Incidence of non-typhoidal salmonella invasive disease: a systematic review and meta-analysis. J Infect.

[13] Park SE, Pak GD, Aaby P (2016). The relationship between invasive nontyphoidal salmonella disease, other bacterial bloodstream infections, and malaria in sub-Saharan Africa. Clin Infect Dis.

[14] Tapia MD, Tennant SM, Bornstein K (2015). Invasive nontyphoidal salmonella infections among children in Mali, 2002–2014: microbiological and epidemiologic features guide vaccine development. Clin Infect Dis.

[15] Still WL, Tapia MD, Tennant SM (2020). Surveillance for invasive salmonella disease in Bamako, Mali, from 2002 to 2018. Clin Infect Dis.

[16] Shahunja KM, Leung DT, Ahmed T (2015). Factors associated with non-typhoidal salmonella bacteremia versus typhoidal salmonella bacteremia in patients presenting for care in an urban diarrheal disease hospital in Bangladesh. PLoS Negl Trop Dis.

[17] Gordon MA, Banda HT, Gondwe M (2002). Non-typhoidal salmonella bacteraemia among HIV-infected Malawian adults: high mortality and frequent recrudescence. AIDS.

[18] Grimont PA, Weill F-X (2007).

[19] Ao TT, Feasey NA, Gordon MA, Keddy KH, Angulo FJ, Crump JA (2015). Global burden of invasive nontyphoidal salmonella disease, 2010(1). Emerg Infect Dis.

[20] Gilchrist JJ, MacLennan CA (2019). Invasive nontyphoidal salmonella disease in Africa. EcoSal Plus.

[21] Kariuki S, Mbae C, Onsare R (2019). Multidrug-resistant nontyphoidal salmonella hotspots as targets for vaccine use in management of infections in endemic settings. Clin Infect Dis.

[22] Kariuki S, Gordon MA, Feasey N, Parry CM (2015). Antimicrobial resistance and management of invasive salmonella disease. Vaccine.

[23] Tack B, Vanaenrode J, Verbakel JY, Toelen J, Jacobs J (2020). Invasive non-typhoidal salmonella infections in sub-Saharan Africa: a systematic review on antimicrobial resistance and treatment. BMC Med.

[24] Balasubramanian R, Im J, Lee J-S (2019). The global burden and epidemiology of invasive non-typhoidal salmonella infections. Hum Vaccines Immunother.

[25] Baliban SM, Lu Y-J, Malley R (2020). Overview of the nontyphoidal and paratyphoidal salmonella vaccine pipeline: current status and future prospects. Clin Infect Dis.

[26] Ouzzani M, Hammady H, Fedorowicz Z, Elmagarmid A (2016). Rayyan—a web and mobile app for systematic reviews. Syst Rev.

[27] Moher D, Liberati A, Tetzlaff J, Altman DG (2009). Preferred reporting items for systematic reviews and meta-analyses: the PRISMA statement. PLoS Med.

[28] EpiGear International Pty Ltd (2016). MetaXL version 5.3. https://www.epigear.com/index_files/metaxl.html.

[29] Barendregt JJ, Doi SA, Lee YY, Norman RE, Vos T (2013). Meta-analysis of prevalence. J Epidemiol Community Health.

[30] Doi SAR, Barendregt JJ, Khan S, Thalib L, Williams GM (2015). Advances in the meta-analysis of heterogeneous clinical trials I: the inverse variance heterogeneity model. Contemp Clin Trials.

[31] Viswanathan M, Patnode CD, Berkman ND (2018). Recommendations for assessing the risk of bias in systematic reviews of health-care interventions. J Clin Epidemiol.

[32] Sterne JAC, Hernán MA, Reeves BC (2016). ROBINS-I: a tool for assessing risk of bias in non-randomised studies of interventions. BMJ.

[33] Munn Z, Moola S, Lisy K, Riitano D, Tufanaru C (2015). Methodological guidance for systematic reviews of observational epidemiological studies reporting prevalence and cumulative incidence data. Int J Evid Based Healthc.

[34] Abu NA, Fadzilah MN, Mariam M (2016). Community-acquired bacteremia in paediatrics: epidemiology, aetiology and patterns of antimicrobial resistance in a tertiary care centre, Malaysia. Med J Malaysia.

[35] Alausa KO, Montefiore D, Sogbetun AO, Ashiru JO, Onile BA, Sobayo E (1977). Septicaemia in the tropics. A prospective epidemiological study of 146 patients with a high case fatality rate. Scand J Infect Dis.

[36] Albert MJ, Bulach D, Alfouzan W (2019). Non-typhoidal salmonella blood stream infection in Kuwait: clinical and microbiological characteristics. PLoS Negl Trop Dis.

[37] Appiah GD, Mpimbaza A, Lamorde M (2021). Salmonella bloodstream infections in hospitalized children with acute febrile illness—Uganda, 2016–2019. Am J Trop Med Hyg.

[38] Bar-Meir M, Raveh D, Yinnon AM, Benenson S, Rudensky B, Schlesinger Y (2005). Non-Typhi salmonella gastroenteritis in children presenting to the emergency department: characteristics of patients with associated bacteraemia. Clin Microbiol Infect.

[39] Bassa A, Parras F, Reina J, Villar E, Gil J, Alomar P (1989). Non-Typhi salmonella bacteraemia. Infection.

[40] Bassat Q, Guinovart C, Sigaúque B (2009). Severe malaria and concomitant bacteraemia in children admitted to a rural Mozambican hospital. Trop Med Int Health.

[41] Blomberg B, Manji KP, Urassa WK (2007). Antimicrobial resistance predicts death in Tanzanian children with bloodstream infections: a prospective cohort study. BMC Infect Dis.

[42] Brent AJ, Oundo JO, Mwangi I, Ochola L, Lowe B, Berkley JA (2006). salmonella bacteremia in Kenyan children. Pediatr Infect Dis J.

[43] Chen P-L, Chang C-M, Wu C-J (2007). Extraintestinal focal infections in adults with nontyphoid salmonella bacteraemia: predisposing factors and clinical outcome. J Intern Med.

[44] Chen S-Y, Weng T-H, Tseng W-P (2018). Value of blood culture time to positivity in identifying complicated nontyphoidal salmonella bacteremia. Diagn Microbiol Infect Dis.

[45] Chou Y-J, Lin H-W, Yang C-J (2016). Risk of recurrent nontyphoid salmonella bacteremia in human immunodeficiency virus-infected patients with short-term secondary prophylaxis in the era of combination antiretroviral therapy. J Microbiol Immunol Infect.

[46] Ciftçi E, Güriz H, Derya Aysev A, Ince E, Erdem B, Doğru U (2004). salmonella bacteraemia in Turkish children: 37 cases seen in a university hospital between 1993 and 2002. Ann Trop Paediatr.

[47] Díez Dorado R, Tagarro García A, Baquero-Artigao F (2004). Non-Typhi salmonella bacteremia in children: an 11-year review. An Pediatr.

[48] Emary K, Moore CE, Chanpheaktra N (2012). Enteric fever in Cambodian children is dominated by multidrug-resistant H58 *Salmonella enterica* serovar Typhi with intermediate susceptibility to ciprofloxacin. Trans R Soc Trop Med Hyg.

[49] Falay D, Kuijpers LMF, Phoba M-F (2016). Microbiological, clinical and molecular findings of non-typhoidal salmonella bloodstream infections associated with malaria, Oriental Province, Democratic Republic of the Congo. BMC Infect Dis.

[50] Feasey NA, Houston A, Mukaka M (2014). A reduction in adult blood stream infection and case fatality at a large African hospital following antiretroviral therapy roll-out. PloS One.

[51] Galanakis E, Bitsori M, Maraki S, Giannakopoulou C, Samonis G, Tselentis Y (2007). Invasive non-typhoidal salmonellosis in immunocompetent infants and children. Int J Infect Dis.

[52] Georgilis K, Prifti H, Kostis E, Petrocheilou-Paschou V (1997). Clinical and microbiologic aspects of nonTyphi salmonella bacteremia in adults. Infect Dis Clin Pract.

[53] Gilks CF, Brindle RJ, Otieno LS (1990). Life-threatening bacteraemia in HIV-1 seropositive adults admitted to hospital in Nairobi, Kenya. Lancet.

[54] Gordon MA, Walsh AL, Chaponda M (2001). Bacteraemia and mortality among adult medical admissions in Malawi-predominance of non-Typhi salmonellae and *Streptococcus pneumoniae*. J Infect.

[55] Gradel KO, Schønheyder HC, Pedersen L, Thomsen RW, Nørgaard M, Nielsen H (2006). Incidence and prognosis of non-typhoid salmonella bacteraemia in Denmark: a 10-year county-based follow-up study. Eur J Clin Microbiol Infect Dis.

[56] Graham SM, Walsh AL, Molyneux EM, Phiri AJ, Molyneux ME (2000). Clinical presentation of non-typhoidal salmonella bacteraemia in Malawian children. Trans R Soc Trop Med Hyg.

[57] Green SD, Cheesbrough JS (1993). Salmonella bacteraemia among young children at a rural hospital in western Zaire. Ann Trop Paediatr.

[58] Guiraud I, Post A, Diallo SN (2017). Population-based incidence, seasonality and serotype distribution of invasive salmonellosis among children in Nanoro, rural Burkina Faso. PLoS One.

[59] Habib AG (2004). A clinical audit of presentation and outcome of salmonella septicaemia. Ann Acad Med Singapore.

[60] Hsu R-B, Lin F-Y (2005). Risk factors for bacteraemia and endovascular infection due to non-typhoid salmonella: a reappraisal. QJM Mon J Assoc Physicians.

[61] Hsu R-B, Chen RJ, Chu S-H (2004). Risk factors for recurrent bacteremia in adult patients with nontyphoid salmonellosis. Am J Med Sci.

[62] Huang I-F, Wagener MM, Hsieh K-S (2004). Nontyphoid salmonellosis in Taiwan children: clinical manifestations, outcome and antibiotic resistance. J Pediatr Gastroenterol Nutr.

[63] Hung C-C, Hung M-N, Hsueh P-R (2007). Risk of recurrent nontyphoid salmonella bacteremia in HIV-infected patients in the era of highly active antiretroviral therapy and an increasing trend of fluoroquinolone resistance. Clin Infect Dis.

[64] Katiyo S, Muller-Pebody B, Minaji M (2019). Epidemiology and outcomes of nontyphoidal salmonella bacteremias from England, 2004 to 2015. J Clin Microbiol.

[65] Kazemi M, Gumpert G, Marks MI (1974). Clinical spectrum and carrier state of nontyphoidal salmonella infections in infants and children. Can Med Assoc J.

[66] Keddy KH, Musekiwa A, Sooka A (2017). Clinical and microbiological features of invasive nontyphoidal salmonella associated with HIV-infected patients, Gauteng Province, South Africa. Medicine (Baltimore).

[67] Kedzierska J, Piatkowska-Jakubas B, Kedzierska A (2008). Clinical presentation of extraintestinal infections caused by non-typhoid salmonella serotypes among patients at the University Hospital in Cracow during an 7-year period. Pol J Microbiol.

[68] Lee SC, Yang PH, Shieh WB, Lasserre R (1994). Bacteremia due to non-Typhi salmonella: analysis of 64 cases and review. Clin Infect Dis.

[69] Lepage P, Bogaerts J, Van Goethem C (1987). Community-acquired bacteraemia in African children. Lancet.

[70] Lepage P, Bogaerts J, Van Goethem C, Hitimana DG, Nsengumuremyi F (1990). Multiresistant *Salmonella* Typhimurium systemic infection in Rwanda. Clinical features and treatment with cefotaxime. J Antimicrob Chemother.

[71] Lester A, Eriksen NH, Nielsen H (1991). Non-typhoid salmonella bacteraemia in Greater Copenhagen 1984 to 1988. Eur J Clin Microbiol Infect Dis.

[72] Li C-W, Chen P-L, Lee N-Y (2012). Non-typhoidal salmonella bacteremia among adults: an adverse prognosis in patients with malignancy. J Microbiol Immunol Infect.

[73] Lin H-W, Hsu H-S, Huang Y-T, Yang C-J, Hsu M-S, Liao C-H (2016). Time to positivity in blood cultures of adults with nontyphoidal salmonella bacteremia. J Microbiol Immunol Infect.

[74] Maltha J, Guiraud I, Kaboré B (2014). Frequency of severe malaria and invasive bacterial infections among children admitted to a rural hospital in Burkina Faso. PLoS One.

[75] Mandal BK, Brennand J (1988). Bacteraemia in salmonellosis: a 15 year retrospective study from a regional infectious diseases unit. BMJ.

[76] Mandomando I, Bassat Q, Sigaúque B (2015). Invasive salmonella infections among children from rural Mozambique, 2001–2014. Clin Infect Dis.

[77] Mohan A, Munusamy C, Tan Y-C (2019). Invasive salmonella infections among children in Bintulu, Sarawak, Malaysian Borneo: a 6-year retrospective review. BMC Infect Dis.

[78] Mtove G, Amos B, von Seidlein L (2010). Invasive salmonellosis among children admitted to a rural Tanzanian hospital and a comparison with previous studies. PloS One.

[79] Muthumbi E, Morpeth SC, Ooko M (2015). Invasive salmonellosis in Kilifi, Kenya. Clin Infect Dis.

[80] Nesbitt A, Mirza NB (1989). Salmonella septicaemias in Kenyan children. J Trop Pediatr.

[81] Nielsen MV, Sarpong N, Krumkamp R (2012). Incidence and characteristics of bacteremia among children in rural Ghana. PloS One.

[82] Noriega LM, Van der Auwera P, Daneau D, Meunier F, Aoun M (1994). salmonella infections in a cancer center. Support Care Cancer.

[83] Ohlsson A, Serenius F (1981). Neonatal septicemia in Riyadh, Saudi Arabia. Acta Paediatr Scand.

[84] Oneko M, Kariuki S, Muturi-Kioi V (2015). Emergence of community-acquired, multidrug-resistant invasive nontyphoidal salmonella disease in rural western Kenya, 2009–2013. Clin Infect Dis.

[85] Patra S, Mukim Y, Varma M, Mukhopadhyay C, Kalwaje Eshwara V (2018). Invasive nontyphoidal salmonella disease in southern India: a 5-year experience from a tertiary care hospital. Turk J Med Sci.

[86] Phoba M-F, De Boeck H, Ifeka BB (2014). Epidemic increase in salmonella bloodstream infection in children, Bwamanda, the Democratic Republic of Congo. Eur J Clin Microbiol Infect Dis.

[87] Phu NH, Day NPJ, Tuan PQ (2020). Concomitant bacteremia in adults with severe falciparum malaria. Clin Infect Dis.

[88] Preveden T, Knezević K, Brkić S, Jelesić Z (2001). Salmonella bacteremia. Med Pregl.

[89] Raucher HS, Eichenfield AH, Hodes HL (1983). Treatment of salmonella gastroenteritis in infants. The significance of bacteremia. Clin Pediatr (Phila).

[90] Roberts FJ (1993). Nontyphoidal, nonparatyphoidal salmonella septicemia in adults. Eur J Clin Microbiol Infect Dis.

[91] Rongkavilit C, Rodriguez ZM, Gómez-Marín O (2000). Gram-negative bacillary bacteremia in human immunodeficiency virus type 1-infected children. Pediatr Infect Dis J.

[92] Ruiz-Contreras J, Ramos JT, Hernández-Sampelayo T (1995). Sepsis in children with human immunodeficiency virus infection. The Madrid HIV Pediatric Infection Collaborative Study Group. Pediatr Infect Dis J.

[93] Seçmeer G, Kanra G, Cemeroğlu AP, Ozen H, Ceyhan M, Ecevit Z (1995). Prognostic factors in *Salmonella typhimurium* septicemia. A 10-year retrospective study. Turk J Pediatr.

[94] Seydi M, Soumare M, Sow AI, Diop BM, Sow PS (2005). Current aspects of salmonella bacteremia cases in the Ibrahima Diop Mar Infectious Diseases clinic, Fann National Hospital Center (Senegal). Med Mal Infect.

[95] Seydi M, Soumare M, Sow AI (2008). Nontyphoidal salmonella bacteremia cases in AIDS patients in a Dakar University Hospital (Senegal). Med Mal Infect.

[96] Shahunja KM, Ahmed T, Hossain MdI (2020). Clinical and laboratory characteristics of children under five hospitalized with diarrhea and bacteremia. PLoS One.

[97] Shimoni Z, Pitlik S, Leibovici L (1999). Nontyphoid salmonella bacteremia: age-related differences in clinical presentation, bacteriology, and outcome. Clin Infect Dis.

[98] Shkalim V, Amir A, Samra Z, Amir J (2012). Characteristics of non-Typhi salmonella gastroenteritis associated with bacteremia in infants and young children. Infection.

[99] Sigaúque B, Roca A, Mandomando I (2009). Community-acquired bacteremia among children admitted to a rural hospital in Mozambique. Pediatr Infect Dis J.

[100] Sirinavin S, Jayanetra P, Thakkinstian A (1999). Clinical and prognostic categorization of extraintestinal nontyphoidal salmonella infections in infants and children. Clin Infect Dis.

[101] Tabu C, Breiman RF, Ochieng B (2012). Differing burden and epidemiology of non-Typhi salmonella bacteremia in rural and urban Kenya, 2006–2009. PloS One.

[102] Tack B, Phoba M-F, Barbé B (2020). Non-typhoidal salmonella bloodstream infections in Kisantu, DR Congo: emergence of O5-negative *Salmonella typhimurium* and extensive drug resistance. PLoS Negl Trop Dis.

[103] Thamlikitkul V, Dhiraputra C, Paisarnsinsup T, Chareandee C (1996). Non-typhoidal salmonella bacteraemia: clinical features and risk factors. Trop Med Int Health.

[104] Tsai M-H, Huang Y-C, Chiu C-H (2007). Nontyphoidal salmonella bacteremia in previously healthy children: analysis of 199 episodes. Pediatr Infect Dis J.

[105] Vaagland H, Blomberg B, Krüger C, Naman N, Jureen R, Langeland N (2004). Nosocomial outbreak of neonatal *Salmonella enterica* serotype Enteritidis meningitis in a rural hospital in northern Tanzania. BMC Infect Dis.

[106] Vandenberg O, Nyarukweba DZ, Ndeba PM (2010). Microbiologic and clinical features of salmonella species isolated from bacteremic children in eastern Democratic Republic of Congo. Pediatr Infect Dis J.

[107] Verani JR, Toroitich S, Auko J (2015). Burden of invasive nontyphoidal salmonella disease in a rural and urban site in Kenya, 2009–2014. Clin Infect Dis.

[108] Vlieghe ER, Phe T, De Smet B (2012). Azithromycin and ciprofloxacin resistance in salmonella bloodstream infections in Cambodian adults. PLoS Negl Trop Dis.

[109] Walsh AL, Phiri AJ, Graham SM, Molyneux EM, Molyneux ME (2000). Bacteremia in febrile Malawian children: clinical and microbiologic features. Pediatr Infect Dis J.

[110] Wang J-Y, Hwang J-J, Hsu C-N, Lin L-C, Hsueh P-R (2006). Bacteraemia due to ciprofloxacin-resistant *Salmonella enterica* serotype Choleraesuis in adult patients at a university hospital in Taiwan, 1996–2004. Epidemiol Infect.

[111] Yen YF, Lin YC, Chen TL (2007). Non-typhoidal salmonella bacteremia in adults. J Microbiol Immunol Infect.

[112] Yen Y-F, Wang F-D, Chiou C-S (2009). Prognostic factors and clinical features of non-typhoid salmonella bacteremia in adults. J Chin Med Assoc.

[113] Yombi JC, Martins L, Vandercam B, Rodriguez-Villalobos H, Robert A (2015). Clinical features and outcome of typhoid fever and invasive non-typhoidal salmonellosis in a tertiary hospital in Belgium: analysis and review of the literature. Acta Clin Belg.

[114] Zaidi E, Bachur R, Harper M (1999). Non-Typhi salmonella bacteremia in children. Pediatr Infect Dis J.

[115] Takem EN, Roca A, Cunnington A (2014). The association between malaria and non-typhoid salmonella bacteraemia in children in sub-Saharan Africa: a literature review. Malar J.

[116] Salomon JA, Haagsma JA, Davis A (2015). Disability weights for the Global Burden of Disease 2013 study. Lancet Glob Health.

[117] GBD 2017 Typhoid and Paratyphoid Collaborators (2019). The global burden of typhoid and paratyphoid fevers: a systematic analysis for the Global Burden of Disease Study 2017. Lancet Infect Dis.

[118] WHO (2020).

[119] Marchello CS, Birkhold M, Crump JA (2020). Complications and mortality of typhoid fever: a global systematic review and meta-analysis. J Infect.

[120] Feasey NA, Cain AK, Msefula CL (2014). Drug resistance in *Salmonella enterica* ser. Typhimurium bloodstream infection, Malawi. Emerg Infect Dis.

[121] Van Puyvelde S, Pickard D, Vandelannoote K (2019). An African *Salmonella typhimurium* ST313 sublineage with extensive drug-resistance and signatures of host adaptation. Nat Commun.

[122] Feasey NA, Hadfield J, Keddy KH (2016). Distinct *Salmonella* Enteritidis lineages associated with enterocolitis in high-income settings and invasive disease in low-income settings. Nat Genet.

[123] Park SE, Pham DT, Pak GD (2021). The genomic epidemiology of multi-drug resistant nontyphoidal salmonella causing invasive disease in sub-Saharan Africa. BMJ Glob Health.

[124] Hardy RJ, Thompson SG (1998). Detecting and describing heterogeneity in meta-analysis. Stat Med.

