# Supplementary material for: Complications and mortality of non-typhoidal salmonella invasive disease: a global systematic review and meta-analysis
Source: Lancet Infect Dis. 2022 May;22(5):692–705. doi: 10.1016/S1473-3099(21)00615-0 (PMC9021030; doi:10.1016/S1473-3099(21)00615-0)

THE LANCET
Infectious Diseases

Supplementary appendix

This appendix formed part of the original submission and has been peer reviewed. We post it as supplied by the authors.

Supplement to: Marchello CS, Birkhold M, Crump JA, on behalf of the Vacc-iNTS consortium collaborators. Complications and mortality of non-typhoidal salmonella invasive disease: a global systematic review and meta-analysis. *Lancet Infect Dis* 2022; published online Jan 31. https://doi.org/10.1016/S1473-3099(21)00615-0.

Supplementary Appendix

Supplement to: Complications and mortality of non-typhoidal *Salmonella* invasive disease: a global systematic review and meta-analysis

Supplementary Appendix A – initial search strategy 21 May 2020

PubMed 21 May 2020

(((((nontyph* OR non-typh* OR "iNTS" OR "salmonella group" OR "salmonellosis" OR typhimurium OR enteritidis OR (salmonella AND Heidelberg) OR (salmonella AND Dublin) OR (salmonella AND Choleraesuis) OR (salmonella AND Newport) OR (salmonella AND Virchow) OR (salmonella AND Concord) OR (salmonella AND Brancaster) OR (salmonella AND freetown) OR (salmonella AND "Infantis") OR (salmonella AND Isangi))))) AND ((mortality OR "morbidity"[All Fields] OR "morbidity"[MeSH Terms] OR died or fatal* or complicat* or meningitis or arthritis or abscess or pneumon* or shock or relapse or recurr* or splen*))) NOT (("Animals, Wild"[MeSH Terms] OR cell OR mouse OR rat OR rodent* OR bat OR bats OR cattle)) AND (hasabstract[text] AND Humans[Mesh])

Search Details

Query Translation:

(((nontyphi[All Fields] OR nontyphodial[All Fields] OR nontyphoid[All Fields] OR nontyphoidal[All Fields] OR nontyphoidalserovars[All Fields] OR nontyphoideal[All Fields] OR nontyphoidic[All Fields] OR nontyphosa[All

Search URL

Result:

2453

Translations:

salmonella	"salmonella"[MeSH Terms] OR "salmonella"[All Fields]
Newport	"Newport"[Journal] OR "newport"[All Fields]
Concord	"Afr Concord"[Journal] OR "concord"[All Fields]
mortality	"mortality"[Subheading] OR "mortality"[All Fields] OR "mortality"[MeSH Terms]
died	"death"[MeSH Terms] OR "death"[All Fields] OR "died"[All Fields]
meningitis	"meningitis"[MeSH Terms] OR "meningitis"[All Fields]
arthritis	"arthritis"[MeSH Terms] OR "arthritis"[All Fields]
abscess	"abscess"[MeSH Terms] OR "abscess"[All Fields]
shock	"shock"[MeSH Terms] OR "shock"[All Fields]
relapse	"recurrence"[MeSH Terms] OR "recurrence"[All Fields] OR "relapse"[All Fields]
cell	"cells"[MeSH Terms] OR "cells"[All Fields] OR "cell"[All Fields]
mouse	"mice"[MeSH Terms] OR "mice"[All Fields] OR "mouse"[All Fields]
rat	"rats"[MeSH Terms] OR "rats"[All Fields] OR "rat"[All Fields]
bat	"Behav Anal Today"[Journal] OR "bat"[All Fields]
bats	"chiroptera"[MeSH Terms] OR "chiroptera"[All Fields] OR "bats"[All Fields]
cattle	"cattle"[MeSH Terms] OR "cattle"[All Fields]
Humans[Mesh]	"humans"[MeSH Terms]

Database:

PubMed

User query:

(((((nontyph* OR non-typh* OR "iNTS" OR "salmonella group" OR "salmonellosis" OR typhimurium OR enteritidis OR (salmonella AND Heidelberg) OR (salmonella AND Dublin) OR (salmonella AND Choleraesuis) OR (salmonella AND Newport) OR (salmonella AND Virchow) OR (salmonella AND Concord) OR (salmonella AND Brancaster) OR (salmonella AND freetown) OR (salmonella AND "Infantis") OR (salmonella AND Isangi))))) AND ((mortality OR "morbidity"[All Fields] OR "morbidity"[MeSH Terms] OR died or fatal* or complicat* or meningitis or arthritis or abscess or pneumon* or shock or relapse or recurr* or splen*))) NOT (("Animals, Wild"[MeSH Terms] OR cell OR mouse OR rat OR rodent* OR bat OR bats OR cattle)) AND (hasabstract[text] AND Humans[Mesh])

Web of Science 21 May 2020

(TS=(mortality OR morbidity OR death OR died or fatal* or complicat* or meningitis or arthritis or abscess or pneumon* or shock or relapse or recurr* or splen*)) AND (TS=(nontyph* OR non-typh* OR "iNTS" OR "salmonella group" OR "salmonellosis" OR typhimurium OR enteritidis) OR TS=(salmonella AND Heidelberg) OR TS=(salmonella AND Dublin) OR TS=(salmonella AND Choleraesuis) OR TS=(salmonella AND Newport) OR TS=(salmonella AND Virchow) OR TS=(salmonella AND Concord) OR TS=(salmonella AND Brancaster) OR TS=(salmonella AND "freetown") OR TS=(salmonella AND "Infantis") OR TS=(salmonella AND Isangi)) NOT (TS=(extract* OR carcass* OR cell OR mouse OR rat OR rodent* OR bat OR bats OR cattle))
Refined by: [excluding] RESEARCH AREAS: (GENETICS HEREDITY OR VETERINARY SCIENCES OR ZOOLOGY OR ENDOCRINOLOGY METABOLISM OR MATHEMATICS OR PLANT SCIENCES OR BUSINESS ECONOMICS OR DERMATOLOGY OR BIOPHYSICS OR MATHEMATICAL COMPUTATIONAL BIOLOGY OR ENGINEERING OR COMPUTER SCIENCE OR MARINE FRESHWATER BIOLOGY OR MATERIALS SCIENCE OR BIODIVERSITY CONSERVATION OR PHYSICS OR METEOROLOGY ATMOSPHERIC SCIENCES OR ANTHROPOLOGY OR GOVERNMENT LAW OR LEGAL MEDICINE OR EDUCATION EDUCATIONAL RESEARCH OR SUBSTANCE ABUSE OR PSYCHIATRY OR POLYMER SCIENCE OR HISTORY OR ELECTROCHEMISTRY OR FORESTRY OR SPORT SCIENCES OR FISHERIES OR GEOGRAPHY OR PUBLIC ADMINISTRATION OR ENERGY FUELS OR INTERNATIONAL RELATIONS) AND [excluding] DOCUMENT TYPES: (ABSTRACT OR LETTER OR MEETING OR EDITORIAL OR BOOK OR PATENT OR NEWS OR REPORT)
Databases= WOS, BCI, CCC, INSPEC, MEDLINE, SCIELO Timespan=All years
Search language=Auto

Set	Results	Save History / Create Alert · Open Saved History	Combine Sets ◯AND ◯OR Combine	Delete Sets Select All ✖ Delete
# 3	2,712	(TS=(mortality OR morbidity OR death OR died or fatal* or complicat* or meningitis or arthritis or abscess or pneumon* or shock or relapse or recurr* or splen*)) AND (TS=(nontyph* OR non-typh* OR "iNTS" OR "salmonella group" OR "salmonellosis" OR typhimurium OR enteritidis) OR TS=(salmonella AND Heidelberg) OR TS=(salmonella AND Dublin) OR TS=(salmonella AND Choleraesuis) OR TS=(salmonella AND Newport) OR TS=(salmonella AND Virchow) OR TS=(salmonella AND Concord) OR TS=(salmonella AND Brancaster) OR TS=(salmonella AND "freetown") OR TS=(salmonella AND "Infantis") OR TS=(salmonella AND Isangi)) NOT (TS=(extract* OR carcass* OR cell OR mouse OR rat OR rodent* OR bat OR bats OR cattle)) **Refined by:** [excluding] **RESEARCH AREAS:** (GENETICS HEREDITY OR VETERINARY SCIENCES OR ZOOLOGY OR ENDOCRINOLOGY METABOLISM OR MATHEMATICS OR PLANT SCIENCES OR BUSINESS ECONOMICS OR DERMATOLOGY OR BIOPHYSICS OR MATHEMATICAL COMPUTATIONAL BIOLOGY OR ENGINEERING OR COMPUTER SCIENCE OR MARINE FRESHWATER BIOLOGY OR MATERIALS SCIENCE OR BIODIVERSITY CONSERVATION OR PHYSICS OR METEOROLOGY ATMOSPHERIC SCIENCES OR ANTHROPOLOGY OR GOVERNMENT LAW OR LEGAL MEDICINE OR EDUCATION EDUCATIONAL RESEARCH OR SUBSTANCE ABUSE OR PSYCHIATRY OR POLYMER SCIENCE OR HISTORY OR ELECTROCHEMISTRY OR FORESTRY OR SPORT SCIENCES OR FISHERIES OR GEOGRAPHY OR PUBLIC ADMINISTRATION OR ENERGY FUELS OR INTERNATIONAL RELATIONS) AND [excluding] **DOCUMENT TYPES:** (ABSTRACT OR LETTER OR MEETING OR EDITORIAL OR BOOK OR PATENT OR NEWS OR REPORT) *Databases= WOS, BCI, CCC, INSPEC, MEDLINE, SCIELO Timespan=All years* *Search language=Auto*	☐	☐
# 2	3,691	(TS=(mortality OR morbidity OR death OR died or fatal* or complicat* or meningitis or arthritis or abscess or pneumon* or shock or relapse or recurr* or splen*)) AND (TS=(nontyph* OR non-typh* OR "iNTS" OR "salmonella group" OR "salmonellosis" OR typhimurium OR enteritidis) OR TS=(salmonella AND Heidelberg) OR TS=(salmonella AND Dublin) OR TS=(salmonella AND Choleraesuis) OR TS=(salmonella AND Newport) OR TS=(salmonella AND Virchow) OR TS=(salmonella AND Concord) OR TS=(salmonella AND Brancaster) OR TS=(salmonella AND "freetown") OR TS=(salmonella AND "Infantis") OR TS=(salmonella AND Isangi)) NOT (TS=(extract* OR carcass* OR cell OR mouse OR rat OR rodent* OR bat OR bats OR cattle)) **Refined by:** [excluding] **RESEARCH AREAS:** (GENETICS HEREDITY OR VETERINARY SCIENCES OR ZOOLOGY OR ENDOCRINOLOGY METABOLISM OR MATHEMATICS OR PLANT SCIENCES OR BUSINESS ECONOMICS OR DERMATOLOGY OR BIOPHYSICS OR MATHEMATICAL COMPUTATIONAL BIOLOGY OR ENGINEERING OR COMPUTER SCIENCE OR MARINE FRESHWATER BIOLOGY OR MATERIALS SCIENCE OR BIODIVERSITY CONSERVATION OR PHYSICS OR METEOROLOGY ATMOSPHERIC SCIENCES OR ANTHROPOLOGY OR GOVERNMENT LAW OR LEGAL MEDICINE OR EDUCATION EDUCATIONAL RESEARCH OR SUBSTANCE ABUSE OR PSYCHIATRY OR POLYMER SCIENCE OR HISTORY OR ELECTROCHEMISTRY OR FORESTRY OR SPORT SCIENCES OR FISHERIES OR GEOGRAPHY OR PUBLIC ADMINISTRATION OR ENERGY FUELS OR INTERNATIONAL RELATIONS) *Databases= WOS, BCI, CCC, INSPEC, MEDLINE, SCIELO Timespan=All years* *Search language=Auto*	☐	☐
# 1	7,880	(TS=(mortality OR morbidity OR death OR died or fatal* or complicat* or meningitis or arthritis or abscess or pneumon* or shock or relapse or recurr* or splen*)) AND (TS=(nontyph* OR non-typh* OR "iNTS" OR "salmonella group" OR "salmonellosis" OR typhimurium OR enteritidis) OR TS=(salmonella AND Heidelberg) OR TS=(salmonella AND Dublin) OR TS=(salmonella AND Choleraesuis) OR TS=(salmonella AND Newport) OR TS=(salmonella AND Virchow) OR TS=(salmonella AND Concord) OR TS=(salmonella AND Brancaster) OR TS=(salmonella AND "freetown") OR TS=(salmonella AND "Infantis") OR TS=(salmonella AND Isangi)) NOT (TS=(extract* OR carcass* OR cell OR mouse OR rat OR rodent* OR bat OR bats OR cattle)) *Databases= WOS, BCI, CCC, INSPEC, MEDLINE, SCIELO Timespan=All years* *Search language=Auto*	☐	☐
			◯AND ◯OR Combine	Select All ✖ Delete

Embase Classic+Embase (1947 to 2020 May 21) + Ovid MEDLINE and Epub Ahead of Print, In-Process & Other Non-Indexed Citations, Daily and Versions (1946 to May 21, 2020)

▼ Search History (42) View Saved

# ▲	Searches	Results	Type	Actions	Annotations
1	(salmonella and typhimurium).af.	81313	Advanced	Display Results More	
2	(salmonella and enteritidis).af.	17788	Advanced	Display Results More	
3	(salmonella and Heidelberg).af.	2561	Advanced	Display Results More	
4	(salmonella and Dublin).af.	2983	Advanced	Display Results More	
5	(salmonella and Choleraesuis).af.	1890	Advanced	Display Results More	
6	(salmonella and newport).af.	1585	Advanced	Display Results More	
7	(salmonella and virchow).af.	882	Advanced	Display Results More	
8	(salmonella and concord).af.	77	Advanced	Display Results More	
9	(salmonella and Brancaster).af.	35	Advanced	Display Results More	
10	(salmonella and freetown).af.	15	Advanced	Display Results More	
11	(salmonella and Infantis).af.	1768	Advanced	Display Results More	
12	(salmonella and Isangi).af.	73	Advanced	Display Results More	
13	remove duplicates from 3	1961	Advanced	Display Results More	
14	remove duplicates from 4	1843	Advanced	Display Results More	
15	remove duplicates from 5	1185	Advanced	Display Results More	
16	remove duplicates from 6	1006	Advanced	Display Results More	
17	remove duplicates from 7	547	Advanced	Display Results More	
18	remove duplicates from 8	51	Advanced	Display Results More	
19	remove duplicates from 9	21	Advanced	Display Results More	
20	remove duplicates from 10	11	Advanced	Display Results More	
21	remove duplicates from 11	1132	Advanced	Display Results More	
22	remove duplicates from 12	43	Advanced	Display Results More	
23	1 or 2 or 13 or 14 or 15 or 16 or 17 or 18 or 19 or 20 or 21 or 22	97931	Advanced	Display Results More	
24	nontyph*.af.	1840	Advanced	Display Results More	
25	non-typh*.af.	2878	Advanced	Display Results More	
26	salmonellosis.af.	30646	Advanced	Display Results More	
27	"iNTS".af.	2187	Advanced	Display Results More	
28	(salmonella adj7 group).af.	2753	Advanced	Display Results More	
29	remove duplicates from 24	1106	Advanced	Display Results More	
30	remove duplicates from 25	1781	Advanced	Display Results More	
31	remove duplicates from 27	1599	Advanced	Display Results More	
32	remove duplicates from 28	1826	Advanced	Display Results More	
33	23 or 26 or 29 or 30 or 31 or 32	119820	Advanced	Display Results More	
34	limit 33 to human	36388	Advanced	Display Results More	
35	limit 34 to journal [Limit not valid in Ovid MEDLINE(R),Ovid MEDLINE(R) Daily Update,Ovid MEDLINE(R) In-Process,Ovid MEDLINE(R) Publisher; records were retained]	36250	Advanced	Display Results More	
36	(mortality or morbidity or death or died or fatal* or complicat* or meningitis or arthritis or abscess or pneumon* or shock or relapse or recurr* or splen*).af.	13331317	Advanced	Display Results More	
37	limit 36 to human	10720398	Advanced	Display Results More	
38	limit 37 to journal [Limit not valid in Ovid MEDLINE(R),Ovid MEDLINE(R) Daily Update,Ovid MEDLINE(R) In-Process,Ovid MEDLINE(R) Publisher; records were retained]	10702725	Advanced	Display Results More	
39	(35 and 38) not (extract* or carcass* or cell or mouse or rat or rodent* or bat or bats or cattle or papillomavirus or "HPV" or aquatic or marine or wild* or "pet" or "pets").af.	7116	Advanced	Display Results More	
40	limit 39 to (article or clinical study or clinical trial, all or clinical trial, phase i or clinical trial, phase ii or clinical trial, phase iii or clinical trial, phase iv or journal article or observational study or randomized controlled trial) [Limit not valid in Embase,Ovid MEDLINE(R),Ovid MEDLINE(R) Daily Update,Ovid MEDLINE(R) In-Process,Ovid MEDLINE(R) Publisher; records were retained]	5532	Advanced	Display Results More	
41	limit 40 to abstracts	4104	Advanced	Display Results More	
42	remove duplicates from 41	3236	Advanced	Display Results More	

Save Remove Combine with: AND OR Deduplicate

Supplementary Appendix B – updated search strategy 4 June 2021

PubMed 4 June 2021

Search: (((((nontyph* OR non-typh* OR "iNTS" OR "salmonella group" OR "salmonellosis" OR typhimurium OR enteritidis OR (salmonella AND Heidelberg) OR (salmonella AND Dublin) OR (salmonella AND Choleraesuis) OR (salmonella AND Newport) OR (salmonella AND Virchow) OR (salmonella AND Concord) OR (salmonella AND Brancaster) OR (salmonella AND freetown) OR (salmonella AND "Infantis") OR (salmonella AND Isangi))))) AND ((mortality OR "morbidity"[All Fields] OR "morbidity"[MeSH Terms] OR died OR fatal* OR complicat* OR meningitis OR arthritis OR abscess OR pneumon* OR shock OR relapse OR recurr* OR splen*))) NOT (("Animals, Wild"[MeSH Terms] OR cell OR mouse OR rat OR rodent* OR bat OR bats OR cattle)) AND (hasabstract[text] AND Humans[Mesh]) AND "2020/05/20 19:29":"2021/06/03 20:15"[Date - MeSH]

History and Search Details

Download Delete

Search	Actions	Details	Query	Results	Time
#1	•••	>	Search: **(((((nontyph* OR non-typh* OR "iNTS" OR "salmonella group" OR "salmonellosis" OR typhimurium OR enteritidis OR (salmonella AND Heidelberg) OR (salmonella AND Dublin) OR (salmonella AND Choleraesuis) OR (salmonella AND Newport) OR (salmonella AND Virchow) OR (salmonella AND Concord) OR (salmonella AND Brancaster) OR (salmonella AND freetown) OR (salmonella AND "Infantis") OR (salmonella AND Isangi))))) AND ((mortality OR "morbidity"[All Fields] OR "morbidity"[MeSH Terms] OR died OR fatal* OR complicat* OR meningitis OR arthritis OR abscess OR pneumon* OR shock OR relapse OR recurr* OR splen*))) NOT (("Animals, Wild"[MeSH Terms] OR cell OR mouse OR rat OR rodent* OR bat OR bats OR cattle)) AND (hasabstract[text] AND Humans[Mesh]) AND "2020/05/20 19:29":"2021/06/03 20:15"[Date - MeSH] - Saved search** Sort by: **Most Recent**	78	20:45:44

Showing 1 to 1 of 1 entries

NCBI Literature Resources MeSH PMC BookShelf

Web of Science 4 June 2020

(TS=(mortality OR morbidity OR death OR died or fatal* or complicat* or meningitis or arthritis or abscess or pneumon* or shock or relapse or recurr* or splen*)) AND (TS=(nontyph* OR non-typh* OR "iNTS" OR "salmonella group" OR "salmonellosis" OR typhimurium OR enteritidis) OR TS=(salmonella AND Heidelberg) OR TS=(salmonella AND Dublin) OR TS=(salmonella AND Choleraesuis) OR TS=(salmonella AND Newport) OR TS=(salmonella AND Virchow) OR TS=(salmonella AND Concord) OR TS=(salmonella AND Brancaster) OR TS=(salmonella AND "freetown") OR TS=(salmonella AND "Infantis") OR TS=(salmonella AND Isangi)) NOT (TS=(extract* OR carcass* OR cell OR mouse OR rat OR rodent* OR bat OR bats OR cattle))
Refined by: [excluding] **RESEARCH AREAS:** (GENETICS HEREDITY OR VETERINARY SCIENCES OR ZOOLOGY OR ENDOCRINOLOGY METABOLISM OR MATHEMATICS OR PLANT SCIENCES OR BUSINESS ECONOMICS OR DERMATOLOGY OR BIOPHYSICS OR MATHEMATICAL COMPUTATIONAL BIOLOGY OR ENGINEERING OR COMPUTER SCIENCE OR MARINE FRESHWATER BIOLOGY OR MATERIALS SCIENCE OR BIODIVERSITY CONSERVATION OR PHYSICS OR METEOROLOGY ATMOSPHERIC SCIENCES OR ANTHROPOLOGY OR GOVERNMENT LAW OR LEGAL MEDICINE OR EDUCATION EDUCATIONAL RESEARCH OR SUBSTANCE ABUSE OR PSYCHIATRY OR POLYMER SCIENCE OR HISTORY OR ELECTROCHEMISTRY OR FORESTRY OR SPORT SCIENCES OR FISHERIES OR GEOGRAPHY OR PUBLIC ADMINISTRATION OR ENERGY FUELS OR INTERNATIONAL RELATIONS) AND [excluding] **DOCUMENT TYPES:** (ABSTRACT OR LETTER OR MEETING OR EDITORIAL OR BOOK OR PATENT OR NEWS OR REPORT)
Databases= WOS, BCI, CCC, INSPEC, KJD, MEDLINE, RSCI, SCIELO, ZOOREC Timespan=2020-2021
Search language=Auto

Set	Results	Save History / Create Alert · Open Saved History	Combine Sets ◯ AND ◯ OR · Combine	Delete Sets · Select All · ✕ Delete
# 3	145	(TS=(mortality OR morbidity OR death OR died or fatal* or complicat* or meningitis or arthritis or abscess or pneumon* or shock or relapse or recurr* or splen*)) AND (TS=(nontyph* OR non-typh* OR "iNTS" OR "salmonella group" OR "salmonellosis" OR typhimurium OR enteritidis) OR TS=(salmonella AND Heidelberg) OR TS=(salmonella AND Dublin) OR TS=(salmonella AND Choleraesuis) OR TS=(salmonella AND Newport) OR TS=(salmonella AND Virchow) OR TS=(salmonella AND Concord) OR TS=(salmonella AND Brancaster) OR TS=(salmonella AND "freetown") OR TS=(salmonella AND "Infantis") OR TS=(salmonella AND Isangi)) NOT (TS=(extract* OR carcass* OR cell OR mouse OR rat OR rodent* OR bat OR bats OR cattle)) **Refined by:** [excluding] **RESEARCH AREAS:** (GENETICS HEREDITY OR VETERINARY SCIENCES OR ZOOLOGY OR ENDOCRINOLOGY METABOLISM OR MATHEMATICS OR PLANT SCIENCES OR BUSINESS ECONOMICS OR DERMATOLOGY OR BIOPHYSICS OR MATHEMATICAL COMPUTATIONAL BIOLOGY OR ENGINEERING OR COMPUTER SCIENCE OR MARINE FRESHWATER BIOLOGY OR MATERIALS SCIENCE OR BIODIVERSITY CONSERVATION OR PHYSICS OR METEOROLOGY ATMOSPHERIC SCIENCES OR ANTHROPOLOGY OR GOVERNMENT LAW OR LEGAL MEDICINE OR EDUCATION EDUCATIONAL RESEARCH OR SUBSTANCE ABUSE OR PSYCHIATRY OR POLYMER SCIENCE OR HISTORY OR ELECTROCHEMISTRY OR FORESTRY OR SPORT SCIENCES OR FISHERIES OR GEOGRAPHY OR PUBLIC ADMINISTRATION OR ENERGY FUELS OR INTERNATIONAL RELATIONS) AND [excluding] **DOCUMENT TYPES:** (ABSTRACT OR LETTER OR MEETING OR EDITORIAL OR BOOK OR PATENT OR NEWS OR REPORT) *Databases= WOS, BCI, CCC, INSPEC, KJD, MEDLINE, RSCI, SCIELO, ZOOREC Timespan=2020-2021* *Search language=Auto*	☐	☐
# 2	151	(TS=(mortality OR morbidity OR death OR died or fatal* or complicat* or meningitis or arthritis or abscess or pneumon* or shock or relapse or recurr* or splen*)) AND (TS=(nontyph* OR non-typh* OR "iNTS" OR "salmonella group" OR "salmonellosis" OR typhimurium OR enteritidis) OR TS=(salmonella AND Heidelberg) OR TS=(salmonella AND Dublin) OR TS=(salmonella AND Choleraesuis) OR TS=(salmonella AND Newport) OR TS=(salmonella AND Virchow) OR TS=(salmonella AND Concord) OR TS=(salmonella AND Brancaster) OR TS=(salmonella AND "freetown") OR TS=(salmonella AND "Infantis") OR TS=(salmonella AND Isangi)) NOT (TS=(extract* OR carcass* OR cell OR mouse OR rat OR rodent* OR bat OR bats OR cattle)) **Refined by:** [excluding] **RESEARCH AREAS:** (GENETICS HEREDITY OR VETERINARY SCIENCES OR ZOOLOGY OR ENDOCRINOLOGY METABOLISM OR MATHEMATICS OR PLANT SCIENCES OR BUSINESS ECONOMICS OR DERMATOLOGY OR BIOPHYSICS OR MATHEMATICAL COMPUTATIONAL BIOLOGY OR ENGINEERING OR COMPUTER SCIENCE OR MARINE FRESHWATER BIOLOGY OR MATERIALS SCIENCE OR BIODIVERSITY CONSERVATION OR PHYSICS OR METEOROLOGY ATMOSPHERIC SCIENCES OR ANTHROPOLOGY OR GOVERNMENT LAW OR LEGAL MEDICINE OR EDUCATION EDUCATIONAL RESEARCH OR SUBSTANCE ABUSE OR PSYCHIATRY OR POLYMER SCIENCE OR HISTORY OR ELECTROCHEMISTRY OR FORESTRY OR SPORT SCIENCES OR FISHERIES OR GEOGRAPHY OR PUBLIC ADMINISTRATION OR ENERGY FUELS OR INTERNATIONAL RELATIONS) *Databases= WOS, BCI, CCC, INSPEC, KJD, MEDLINE, RSCI, SCIELO, ZOOREC Timespan=2020-2021* *Search language=Auto*	☐	☐
# 1	291	(TS=(mortality OR morbidity OR death OR died or fatal* or complicat* or meningitis or arthritis or abscess or pneumon* or shock or relapse or recurr* or splen*)) AND (TS=(nontyph* OR non-typh* OR "iNTS" OR "salmonella group" OR "salmonellosis" OR typhimurium OR enteritidis) OR TS=(salmonella AND Heidelberg) OR TS=(salmonella AND Dublin) OR TS=(salmonella AND Choleraesuis) OR TS=(salmonella AND Newport) OR TS=(salmonella AND Virchow) OR TS=(salmonella AND Concord) OR TS=(salmonella AND Brancaster) OR TS=(salmonella AND "freetown") OR TS=(salmonella AND "Infantis") OR TS=(salmonella AND Isangi)) NOT (TS=(extract* OR carcass* OR cell OR mouse OR rat OR rodent* OR bat OR bats OR cattle)) *Databases= WOS, BCI, CCC, INSPEC, KJD, MEDLINE, RSCI, SCIELO, ZOOREC Timespan=2020-2021* *Search language=Auto*	☐	☐
			◯ AND ◯ OR · Combine	Select All · ✕ Delete

Clarivate © 2021 Clarivate Copyright notice Terms of use Privacy statement Cookie policy

Embase Classic+Embase (1947 to 2020 May 21) + Ovid MEDLINE and Epub Ahead of Print, In-Process, In-Data-Review & Other Non-Indexed Citations, Daily and Versions (2020 to June 4, 2021)

Ovid® | Wolters Kluwer

My Account | OvidPV | University of Otago Library | Help | Feedback | Logged in as Christian Marchello | Logoff | Electronic Access Help

Search | Journals | Books | Multimedia | My Workspace | What's New

▼ Search History (43) | View Saved

# ▲	Searches	Results	Type	Actions	Annotations
1	(salmonella and typhimurium).af.	83916	Advanced	Display Results More	
2	(salmonella and enteritidis).af.	18591	Advanced	Display Results More	
3	(salmonella and Heidelberg).af.	2638	Advanced	Display Results More	
4	(salmonella and Dublin).af.	3063	Advanced	Display Results More	
5	(salmonella and Choleraesuis).af.	1946	Advanced	Display Results More	
6	(salmonella and newport).af.	1671	Advanced	Display Results More	
7	(salmonella and virchow).af.	920	Advanced	Display Results More	
8	(salmonella and concord).af.	84	Advanced	Display Results More	
9	(salmonella and Brancaster).af.	36	Advanced	Display Results More	
10	(salmonella and freetown).af.	15	Advanced	Display Results More	
11	(salmonella and Infantis).af.	1909	Advanced	Display Results More	
12	(salmonella and Isangi).af.	77	Advanced	Display Results More	
13	remove duplicates from 3	2009	Advanced	Display Results More	
14	remove duplicates from 4	1905	Advanced	Display Results More	
15	remove duplicates from 5	1225	Advanced	Display Results More	
16	remove duplicates from 6	1070	Advanced	Display Results More	
17	remove duplicates from 7	579	Advanced	Display Results More	
18	remove duplicates from 8	56	Advanced	Display Results More	
19	remove duplicates from 9	22	Advanced	Display Results More	
20	remove duplicates from 10	11	Advanced	Display Results More	
21	remove duplicates from 11	1250	Advanced	Display Results More	
22	remove duplicates from 12	46	Advanced	Display Results More	
23	1 or 2 or 13 or 14 or 15 or 16 or 17 or 18 or 19 or 20 or 21 or 22	101209	Advanced	Display Results More	
24	nontyph*.af.	1981	Advanced	Display Results More	
25	non-typh*.af.	3143	Advanced	Display Results More	
26	salmonellosis.af.	31629	Advanced	Display Results More	
27	"iNTS".af.	2342	Advanced	Display Results More	
28	(salmonella adj7 group).af.	2862	Advanced	Display Results More	
29	remove duplicates from 24	1209	Advanced	Display Results More	
30	remove duplicates from 25	1982	Advanced	Display Results More	
31	remove duplicates from 27	1704	Advanced	Display Results More	
32	remove duplicates from 28	1912	Advanced	Display Results More	
33	23 or 26 or 29 or 30 or 31 or 32	123853	Advanced	Display Results More	
34	limit 33 to human	37763	Advanced	Display Results More	
35	limit 34 to journal [Limit not valid in Ovid MEDLINE(R),Ovid MEDLINE(R) Daily Update,Ovid MEDLINE(R) PubMed not MEDLINE,Ovid MEDLINE(R) In-Process,Ovid MEDLINE(R) Publisher; records were retained]	37613	Advanced	Display Results More	
36	(mortality or morbidity or death or died or fatal* or complicat* or meningitis or arthritis or abscess or pneumon* or shock or relapse or recurr* or splen*).af.	14208986	Advanced	Display Results More	
37	limit 36 to human	11454025	Advanced	Display Results More	
38	limit 37 to journal [Limit not valid in Ovid MEDLINE(R),Ovid MEDLINE(R) Daily Update,Ovid MEDLINE(R) PubMed not MEDLINE,Ovid MEDLINE(R) In-Process,Ovid MEDLINE(R) Publisher; records were retained]	11434306	Advanced	Display Results More	
39	(35 and 38) not (extract* or carcass* or cell or mouse or rat or rodent* or bat or bats or cattle or papillomavirus or "HPV" or aquatic or marine or wild* or "pet" or "pets").af.	7330	Advanced	Display Results More	
40	limit 39 to (article or clinical study or clinical trial, all or clinical trial, phase i or clinical trial, phase ii or clinical trial, phase iii or clinical trial, phase iv or journal article or observational study or randomized controlled trial) [Limit not valid in Embase,Ovid MEDLINE(R),Ovid MEDLINE(R) Daily Update,Ovid MEDLINE(R) PubMed not MEDLINE,Ovid MEDLINE(R) In-Process,Ovid MEDLINE(R) Publisher; records were retained]	5736	Advanced	Display Results More	
41	limit 40 to abstracts	4280	Advanced	Display Results More	
42	remove duplicates from 41	3392	Advanced	Display Results More	
43	limit 42 to yr="2020 -Current"	146	Advanced	Display Results More	

Contract

Save | Remove | Combine with: AND | OR | Deduplicate

Save All | Edit | Create RSS | View Saved | Email All Search History | Copy Search History Link | Copy Search History Details

Supplementary Appendix C - iNTS mortality systematic review data abstraction

# iNTS mortality systematic review data abstraction

* Required

1. Name of reviewer *

Mark only one oval.

- ( ) Christian Marchello
- ( ) Megan Birkhold
- ( ) John Crump

2. First author's last name *

____

3. Publication year *

____

4. PubMed ID *

____

Skip to question 5

Study characteristics

5. Study country *

____

6. Study locality (city, district, or province) *

____

7. UN region *

https://unstats.un.org/unsd/methodology/m49/

Mark only one oval.

- ( ) Africa *Skip to question 13*
- ( ) Americas *Skip to question 14*
- ( ) Asia *Skip to question 15*
- ( ) Europe *Skip to question 16*
- ( ) Oceania *Skip to question 17*

Study characteristics

8. Study setting *

Mark only one oval.

- ( ) Hospital-based
- ( ) Community-based
- ( ) Laboratory-based
- ( ) National surveillance
- ( ) Other: ____________________

9. Date data collection began *

If no specific day provided, use the first of the month; if only a year is provided, enter January 1

____

Example: January 7, 2019

10. Date data collection ended *

If no specific day provided, use the last day of the month; if only a year is provided, enter December 31

____

Example: January 7, 2019

11. Normally sterile site cultured *

Check all that apply.

- [ ] Blood
- [ ] Bone marrow
- [ ] CSF
- [ ] Abscess
- [ ] Synovial fluid

Other: [ ] ____________________

12. Inclusion age group *

Based on inclusion criteria or age range data provided in results

Mark only one oval.

- ( ) Children only (<=15y)
- ( ) Adults only (>15y)
- ( ) Mixed ages

Skip to question 18

Africa sub-regions

13.

Mark only one oval.

- ( ) Eastern Africa
- ( ) Middle Africa
- ( ) Northern Africa
- ( ) Southern Africa
- ( ) Western Africa

Skip to question 8

Americas sub-regions

14.

Mark only one oval.

- Caribbean
- Central America
- Northern America
- South America

Skip to question 8

Asia sub-regions

15.

Mark only one oval.

- Central Asia
- Eastern Asia
- South-eastern Asia
- Southern Asia
- Western Asia

Skip to question 8

Europe sub-regions

16.

Mark only one oval.

- Eastern Europe
- Northern Europe
- Southern Europe
- Western Europe

Skip to question 8

Oceania sub-regions

17.

Mark only one oval.

- Australia and New Zealand
- Melanesia
- Micronesia
- Polynesia

Skip to question 8

Exclude

Should article be excluded after reviewing the data?

18. Exclude?

Mark only one oval.

- Yes *Skip to question 52*
- No *Skip to question 19*

Mortality data

19. Is data on mortality provided?

Mark only one oval.

- Yes *Skip to question 20*
- No *Skip to question 37*

Skip to question 19

Mortality data

20. Which NTS serovar is mortality data available? *

Mark only one oval.

- NTS only (does not stratify by serovar) *Skip to question 21*
- Salmonella Typhimurium *Skip to question 23*
- Salmonella Enteritidis *Skip to question 25*
- Salmonella Heidelberg *Skip to question 27*
- Salmonella Dublin *Skip to question 29*
- Salmonella Newport *Skip to question 31*
- Salmonella Choleraesuis *Skip to question 33*
- Other *Skip to question 35*

Mortality data

21. Cases of NTS only (does not stratify by serovar)

Number cultured from normally sterile sites

22. Number of deaths with NTS

Skip to question 37

Mortality data

23. Cases of Salmonella Typhimurium

Number cultured from normally sterile sites

24. Number of deaths with Salmonella Typhimurium

Skip to question 37

Mortality data

25. Cases of Salmonella Enteritidis

Number cultured from normally sterile sites

26. Number of deaths with Salmonella Enteritidis

Skip to question 37

Mortality data

27. Cases of Salmonella Heidelberg

Number cultured from normally sterile sites

28. Number of deaths with Salmonella Heidelberg

Skip to question 37

Mortality data

29. Cases of Salmonella Dublin
Number cultured from normally sterile sites

30. Number of deaths with Salmonella Dublin

Skip to question 37

Mortality data

31. Cases of Salmonella Newport
Number cultured from normally sterile sites

32. Number of deaths with Salmonella Newport

Skip to question 37

Mortality Data

33. Cases of Salmonella Choleraesuis
Number cultured from normally sterile sites

34. Number of deaths with Salmonella Choleraesuis

Skip to question 37

Mortality data

35. Cases of other non-typhoidal Salmonella

Specify serovar and number cultured from normally sterile sites

36. Number of deaths with other NTS

Skip to question 37

Complications data

37. Is data on complications provided?

Mark only one oval.

- ( ) Yes *Skip to question 38*
- ( ) No *Skip to question 39*

Complications data

Match serovar used in mortality data. If no mortality data, list serovar. Document meningitis, abscess, osteomyelitis, other focal infections, and relapse even if not described by authors as a complication

38. Complications

Complication 1 (number/total culture confirmed cases); Complication 2

HIV data

39. Is mortality stratified by HIV status?

Mark only one oval.

- ( ) Yes *Skip to question 41*
- ( ) No *Skip to question 40*

Malaria data

40. Is mortality stratified by malaria status?

Mark only one oval.

- ( ) Yes *Skip to question 46*
- ( ) No *Skip to question 51*

HIV data

41. HIV confirmation method

Check all that apply.

- [ ] Molecular (PCR, nucleic acid)
- [ ] Serology
- [ ] Not provided

42. Cases of NTS+ and HIV+

____

43. NTS+ and HIV+ deaths

____

44. Cases of NTS+ and HIV-

____

45. NTS+ and HIV- deaths

____

Skip to question 40

Malaria data

46. Malaria confirmation method

Check all that apply.

- [ ] Microscopic (thin or thick blood smear)
- [ ] Serology (includes RDT)
- [ ] Molecular
- [ ] Not provided

47. Cases of NTS+ and malaria+

____

48. NTS+ and malaria+ deaths

____

49. Cases of NTS+ and malaria-

50. NTS+ and malaria- deaths

Scan of reference list

51. List of references needing check

Based on context in which citation was used and the title of article

Notes

52. Notes on the article, if necessary

This content is neither created nor endorsed by Google.

Google Forms

Supplementary Appendix D1. Bias assessment definitions

Grading	Definition
L	Low risk of bias
M	Moderate risk of bias
H	High risk of bias
U	Unknown or unavailable to assess bias

Domain question	Explanation	Low risk definition	High risk definition
Study design (L/H/U)	Type of study design used to ascertain NTS deaths and complications.	Active population- or household-based surveillance, prospective observational	Passive surveillance, retrospective studies, case-control, medical or laboratory records review
Study setting (L/H/U)	Hospital/inpatient or community/outpatient	Community or outpatient based recruitment	Admitted or inpatient hospital-based recruitment
Patient selection (L/H/U)	Were specific populations targeted or were all patients were eligible for recruitment	Described a systematic collection of blood culture among eligible patients	Targeted a population (e.g. children, only adults, or groups such as HIV only) or non-NTS clinical syndrome (e.g. respiratory)
Final year of data collection (L/M/H)	More confidence in newer studies having more robust recruitment and analytical methods	Data collection from 2010 through 2020	Data collection 2000 and older
Definition for complications (L/H/U)	Did the authors clearly define what criteria were used to classify a complication?	Clearly attributed a complication to NTS	Unclear or possibly mixed clinical diagnosis or syndromes as a complication

Patient follow up (L/H/U)	Were patients lost to follow up and was an appropriate follow up period defined for attributing a death to NTS	All patients were followed and the period for ascertaining mortality was <=7 days or authors explicitly describe deaths are directly attributed to NTS	Loss to follow up and mortality period was either >7 days (e.g. 30-day mortality) or unclear in whether a death was a result of NTS
Microbiology methods (L/H/U)	Were microbiology methods fully presented, including blood culture volume and contamination. Clearly defined how infection was attributed to NTS and not Salmonella Typhi or Paratyphi A, B, or C.	Appropriate microbiological methods presented for ruling out typhoidal serotypes. Described blood culture volume and contamination.	Unclear or unconventional microbiological methods. No information on blood culture volumes and contamination.
**Domain subtotal**			
0 of 3 H risk	Low risk		
1 of 3 H risk	Moderate risk		
>1 H risk	High risk		
**Overall risk of bias**			
Both subtotals low risk	Low risk		
1 of 2 high risk	Moderate risk		
Both subtotals high risk	High risk		

Supplementary Appendix D2. Bias assessment scores

			Selection and recruitment				Domain subtotal	Measurement and reporting bias			Domain subtotal	
**Author**	**Year**	**PubMed ID**	Study design (L/H/U)	Study setting (L/H/U)	Patient selection (L/H/U)	Final year of data collection (L/M/H)	L/H/U	Definition for complications (L/H/U)	Patient follow up (L/H/U)	Microbiology methods (L/H/U)	L/H/U	**Overall assessment of risk of bias (L/M/H)**
Abu	2016	27495884	H	H	H	L	H	U	H	L	H	**H**
Alausa	1977	410093	L	H	H	H	H	U	H	H	H	**H**
Albert	2019	30986214	L	H	L	L	M	U	H	H	H	**M**
Appiah	2021	33999850	L	H	H	L	H	U	H	L	M	**M**
Bar-Meir	2005	16008618	H	H	H	M	H	L	L	H	M	**H**
Bassa	1989	2689346	H	H	L	H	H	L	L	H	M	**H**
Bassat	2009	19552643	H	H	H	M	H	U	H	H	H	**H**
Blomberg	2007	17519011	L	H	H	M	H	U	H	L	H	**H**
Brent	2006	16511385	L	H	H	M	H	L	L	H	M	**H**
Chen	2007	17222172	H	H	H	M	H	L	H	H	H	**H**
Chen	2018	29526450	H	H	H	L	H	H	H	H	H	**H**
Chou	2016	26316009	H	H	H	L	H	H	H	H	H	**H**
Ciftci	2004	15005970	H	H	H	M	H	U	H	H	H	**H**
Diez Dorado	2004	15033112	H	H	H	M	H	L	H	H	H	**H**
Emary	2012	23122884	H	H	H	L	H	L	L	H	M	**M**
Falay	2016	27286886	L	H	H	L	H	H	H	H	H	**H**
Feasey	2014	24643091	L	H	H	L	H	U	L	L	L	**M**
Galanakis	2007	16564718	L	H	H	M	H	U	L	H	H	**H**
Georgilis	1997	None	H	H	L	H	H	U	L	H	H	**H**
Gilks	1990	1975046	L	H	H	H	H	U	H	L	H	**H**
Gordon	2001	11243753	L	H	H	H	H	U	L	L	L	**M**
Gradel	2006	16534567	H	H	L	M	H	U	H	H	H	**H**
Graham	2000	10975008	L	H	H	H	H	U	H	H	H	**H**
Green	1993	7681645	L	H	H	H	H	H	L	L	M	**H**

Guiraud	2017	28692655	L	L	H	L	M	L	L	L	L	**M**
Habib	2004	15608832	H	H	L	M	H	L	H	H	H	**H**
Hsu	2004	15599326	H	H	H	M	H	U	H	H	H	**H**
Hsu	2005	16203825	H	H	H	M	H	U	H	H	H	**H**
Huang	2004	15097441	L	H	H	M	H	L	H	H	H	**H**
Hung	2007	17682981	L	H	H	M	H	L	H	H	H	**H**
Katiyo	2019	30381422	H	H	L	L	H	L	H	H	H	**H**
Kazemi	1974	4857958	L	H	H	H	H	L	H	H	H	**H**
Keddy	2017	28353576	H	L	L	L	M	U	H	H	H	**H**
Kedzierska	2008	18610655	H	H	L	M	H	U	L	H	H	**H**
Lee	1994	7803634	L	H	L	H	M	L	H	H	H	**H**
Lepage	1987	2885453	L	L	H	H	M	L	H	H	H	**M**
Lepage	1990	2228845	H	H	H	H	H	H	H	H	H	**H**
Lester	1991	1915383	L	H	L	H	M	L	H	H	H	**H**
Li	2012	22209686	H	H	H	M	H	L	H	H	H	**H**
Lin	2016	25311404	L	H	H	L	H	U	H	H	H	**H**
Maltha	2014	24551225	L	H	H	L	H	U	H	L	H	**H**
Mandal	1988	3145067	H	H	L	H	H	U	H	H	H	**H**
Mandomando	2015	26449950	L	H	H	M	H	U	H	L	H	**H**
Mohan	2019	30999894	H	H	H	L	H	L	L	H	M	**M**
Mtove	2010	20168998	L	H	H	M	H	U	H	L	H	**H**
Muthumbi	2015	26449944	H	H	L	L	H	U	H	H	H	**H**
Nesbitt	1989	2651690	L	H	H	H	H	L	H	L	H	**H**
Nielsen	2012	22970162	L	H	H	M	H	U	H	L	H	**H**
Noriega	1994	8156265	H	H	H	H	H	H	L	H	H	**H**
Ohlsson	1981	7034458	L	H	H	H	H	U	H	H	H	**H**
Oneko	2015	26449946	L	H	H	L	H	H	H	L	H	**H**
Patra	2018	30384571	H	H	L	L	H	H	H	H	H	**H**
Phoba	2014	23975545	H	H	H	L	H	U	H	L	H	**H**
Phu	2020	32107527	L	H	H	M	H	U	L	L	L	**M**
Phu Huong Lan	2016	27513951	H	H	L	L	H	U	H	L	H	**H**
Preveden	2001	11905187	L	H	H	H	H	L	L	H	M	**M**

Raucher	1983	6883890	L	L	H	H	M	L	L	H	M	**M**
Roberts	1993	8508819	H	H	L	H	H	H	H	H	H	**H**
Rongkavilit	2000	10693998	H	H	H	H	H	U	H	H	H	**H**
Ruiz-Contreras	1995	7667058	H	H	H	H	H	L	L	H	M	**H**
Secmeer	1995	7502360	H	H	H	H	H	H	H	H	H	**H**
Seydi	2005	15695029	H	H	L	M	H	H	H	H	H	**H**
Seydi	2008	18093773	H	H	L	M	H	H	H	H	H	**H**
Shahunja	2020	33264364	H	H	H	L	H	U	L	L	L	**M**
Shahunja	2015	26361076	H	H	L	L	H	U	L	H	M	**H**
Shimoni	1999	10825045	H	H	L	H	H	L	L	H	M	**M**
Shkalim	2012	22161258	H	H	H	L	H	L	L	H	M	**M**
Sigauque	2009	19131902	L	H	H	M	H	U	H	L	M	**H**
Sirinavin	1999	10524956	H	H	H	H	H	H	H	H	H	**H**
Still	2020	32725229	L	H	H	M	H	U	L	L	L	**M**
Tabu	2012	22363591	L	L	L	L	L	U	H	H	H	**M**
Tack	2020	32240161	L	H	L	L	M	L	U	L	L	**M**
Thamlikitkul	1996	8765451	H	H	H	H	H	H	H	H	H	**H**
Tsai	2007	17901796	H	H	H	M	H	L	L	H	M	**H**
Vaagland	2004	15367335	L	H	H	M	H	U	L	H	H	**H**
Vandenberg	2010	20104200	H	H	H	M	H	U	H	H	H	**H**
Verani	2015	26449945	L	L	L	L	L	U	H	L	H	**M**
Vlieghe	2012	23272255	L	H	H	L	H	U	H	L	H	**H**
Walsh	2000	10783021	L	H	H	H	H	H	H	L	H	**H**
Wang	2006	16569283	H	H	H	M	H	H	H	H	H	**H**
Yen	2007	17639163	H	H	L	M	H	H	L	H	H	**H**
Yen	2009	19686996	L	H	H	M	H	H	H	H	H	**H**
Yombi	2015	25819116	H	H	L	L	H	H	L	H	H	**H**
Zaidi	1999	10608627	H	H	H	H	H	H	L	H	H	**H**

Supplementary Table S1 - study characteristics of included articles

First author's last name	Publication year	PubMed ID	Study country	Study locality (city, district, or province)	UN region	UN sub-region*	Study setting	Date data collection began	Date data collection ended
Abu	2016	27495884	Malaysia	Gombak District	Asia	South-eastern Asia	Hospital-based	1-Jan-2001	31-Dec-2011
Alausa	1977	410093	Nigeria	Ibadan	Africa	Western Africa	Hospital-based	1-Jan-1976	30-Jun-1976
Albert	2019	30986214	Kuwait	Kuwait City	Asia	Western Asia	Hospital-based	1-Apr-2013	31-May-2016
Appiah	2021	33999850	Uganda	Apac, Tororo, Arua, Kabale, Jinja, and Mubende	Africa	Eastern Africa	Hospital-based	1-Jul-2016	31-Jan-2019
Bar-Meir	2005	16008618	Israel	Jerusalem	Asia	Western Asia	Hospital-based	1-Jan-1990	31-Dec-2001
Bassa	1989	2689346	Spain	Palma de Mallorca	Europe	Southern Europe	Hospital-based	1-Jan-1979	30-Apr-1988
Bassat	2009	19552643	Mozambique	Manhiça	Africa	Eastern Africa	Hospital-based	1-Jun-2003	31-May-2007
Blomberg	2007	17519011	Tanzania	Dar es Salaam	Africa	Eastern Africa	Hospital-based	1-Aug-2001	31-Aug-2002
Brent	2006	16511385	Kenya	Kilifi	Africa	Eastern Africa	Hospital-based	1-Aug-1998	31-Jul-2002
Chen	2007	17222172	Taiwan	Tainan	Asia	Eastern Asia	Hospital-based	1-Jan-1999	30-Jun-2005
Chen	2018	29526450	Taiwan	Taipei	Asia	Eastern Asia	Hospital-based	1-Jan-2013	30-Jun-2015
Chou	2016	26316009	Taiwan	Taipei and New Taipei City	Asia	Eastern Asia	Hospital-based	1-Jan-2006	30-Jun-2014
Ciftci	2004	15005970	Turkey	Ankara	Asia	Western Asia	Hospital-based	1-Jan-1993	31-Dec-2002
Diez Dorado	2004	15033112	Spain	Madrid	Europe	Southern Europe	Hospital-based	1-Jan-1992	31-Dec-2002
Emary	2012	23122884	Cambodia	Siem Reap	Asia	South-eastern Asia	Hospital-based	1-Jan-2007	31-Dec-2011
Falay	2016	27286886	Democratic Republic of the Congo	Oriental Province	Africa	Middle Africa	Hospital-based	1-May-2009	31-May-2014
Feasey	2014	24643091	Malawi	Blantyre	Africa	Eastern Africa	Hospital-based	1-Jan-2009	31-Dec-2010
Galanakis	2007	16564718	Greece	Heraklion	Europe	Southern Europe	Hospital-based	1-Jan-1993	31-Dec-2002
Georgilis	1997	None	Greece	Athens	Europe	Southern Europe	Hospital-based	1-Jan-1987	31-Dec-1996
Gilks	1990	1975046	Kenya	Nairobi	Africa	Eastern Africa	Hospital-based	30-Nov-1988	15-May-1989
Gordon	2001	11243753	Malawi	Blantyre	Africa	Eastern Africa	Hospital-based	1-Dec-1997	30-Nov-1998
Gradel	2006	16534567	Denmark	North Jutland County	Europe	Northern Europe	National surveillance	1-Jan-1994	31-Dec-2003

Graham	2000	10975008	Malawi	Blantyre	Africa	Eastern Africa	Hospital-based	14-Feb-1996	30-Apr-1998
Green	1993	7681645	Democratic Republic of the Congo	Kimpese	Africa	Middle Africa	Hospital-based	1-Jan-1982	31-Dec-1986
Guiraud	2017	28692655	Burkina Faso	Nanoro	Africa	Western Africa	Hospital-based	13-May-2013	12-May-2014
Habib	2004	15608832	Singapore	Singapore	Asia	South-eastern Asia	Hospital-based	1-Apr-2001	31-Mar-2003
Hsu	2004	15599326	Taiwan	Taipei	Asia	Eastern Asia	Hospital-based	1-Sep-1984	31-Dec-2003
Hsu	2005	16203825	Taiwan	Taipei	Asia	Eastern Asia	Hospital-based	1-Sep-1984	30-Sep-2004
Huang	2004	15097441	Taiwan	Kaohsiung	Asia	Eastern Asia	Hospital-based	1-Jan-1996	31-Jan-2002
Hung	2007	17682981	Taiwan	Taipei	Asia	Eastern Asia	Hospital-based	1-Jun-1994	30-Jun-2006
Katiyo	2019	30381422	United Kingdom	England	Europe	Northern Europe	National surveillance	1-Jan-2004	31-Dec-2015
Kazemi	1974	4857958	Canada	Montreal	Americas	Northern America	Hospital-based	1-Jun-1971	30-Jun-1972
Keddy	2017	28353576	South Africa	Gauteng Province	Africa	Southern Africa	Laboratory-based	1-Jan-2003	31-Dec-2013
Kedzierska	2008	18610655	Poland	Cracow	Europe	Eastern Europe	Hospital-based	1-Jan-2000	31-Dec-2006
Lee	1994	7803634	Taiwan	Taoyuan	Asia	Eastern Asia	Hospital-based	1-Sep-1985	31-Aug-1990
Lepage	1990	2228845	Rwanda	Kigali	Africa	Eastern Africa	Hospital-based	1-Mar-1982	31-Dec-1987
Lepage	1987	2885453	Rwanda	Kigali	Africa	Eastern Africa	Community-based	16-Oct-1984	15-Oct-1985
Lester	1991	1915383	Denmark	Copenhagen	Europe	Northern Europe	Laboratory-based	1-Jan-1984	31-Dec-1988
Li	2012	22209686	Taiwan	Tainan	Asia	Eastern Asia	Hospital-based	1-Jan-2000	31-Dec-2009
Lin	2016	25311404	Taiwan	New Taipei City	Asia	Eastern Asia	Hospital-based	1-Jan-2010	31-Dec-2012
Maltha	2014	24551225	Burkina Faso	Nanoro	Africa	Western Africa	Hospital-based	1-Jul-2012	31-Jul-2013
Mandal	1988	3145067	United Kingdom - England	Manchester	Europe	Northern Europe	Hospital-based	1-Jan-1975	31-Dec-1983
Mandomando	2015	26449950	Mozambique	Manhiça	Africa	Eastern Africa	Hospital-based	1-Jan-2001	31-Dec-2001
Mohan	2019	30999894	Malaysia	Bintulu	Asia	South-eastern Asia	Hospital-based	1-Jan-2011	31-Dec-2016
Mtove	2010	20168998	Tanzania	Muheza	Africa	Eastern Africa	Hospital-based	1-Mar-2008	28-Feb-2009
Muthumbi	2015	26449944	Kenya	Kilifi	Africa	Eastern Africa	Hospital-based	1-Aug-1998	31-Dec-2014
Nesbitt	1989	2651690	Kenya	Nairobi	Africa	Eastern Africa	Hospital-based	1-Jul-1986	30-Nov-1986
Nielsen	2012	22970162	Ghana	Ashanti region	Africa	Western Africa	Hospital-based	1-Sep-2007	31-Jul-2009
Noriega	1994	8156265	Belgium	Brussels	Europe	Western Europe	Hospital-based	1-Jan-1975	31-Dec-1990
Ohlsson	1981	7034458	Saudi Arabia	Riyadh	Asia	Western Asia	Hospital-based	1-Nov-1976	31-Oct-1980
Oneko	2015	26449946	Kenya	Siaya District	Africa	Eastern Africa	Hospital-based	1-Jul-2009	31-Dec-2013

Patra	2018	30384571	India	Manipal	Asia	Southern Asia	Hospital-based	1-Jan-2012	31-Dec-2016
Phoba	2014	23975545	Democratic Republic of the Congo	Bwamanda	Africa	Middle Africa	Hospital-based	1-Nov-2011	31-May-2012
Phu	2020	32107527	Vietnam	Ho Chi Minh City	Asia	South-eastern Asia	Hospital-based	1-Jan-1991	31-Dec-2003
Phu Huong Lan	2016	27513951	Vietnam	Ho Chi Minh City	Asia	South-eastern Asia	Hospital-based	1-Jan-2008	30-Jun-2013
Preveden	2001	11905187	Serbia	Novi Sad	Europe	Southern Europe	Hospital-based	1-Jan-1991	31-Dec-1998
Raucher	1983	6883890	United States	New York	Americas	Northern America	Hospital-based	1-Jan-1981	31-Dec-1981
Roberts	1993	8508819	Canada	Vancouver	Americas	Northern America	Hospital-based	1-Jan-1980	31-Jan-1992
Rongkavilit	2000	10693998	United States	Miami, FL	Americas	Northern America	Hospital-based	1-Apr-1980	31-Mar-1997
Ruiz-Contreras	1995	7667058	Spain	Madrid	Europe	Southern Europe	Hospital-based	1-Jan-1984	31-Jan-1994
Secmeer	1995	7502360	Turkey	Ankara	Asia	Western Asia	Hospital-based	1-Aug-1982	30-Sep-1992
Seydi	2005	15695029	Senegal	Dakar	Africa	Western Africa	Hospital-based	1-Jan-1996	31-Dec-2003
Seydi	2008	18093773	Senegal	Dakar	Africa	Western Africa	Hospital-based	1-Jan-1996	31-Dec-2005
Shahunja	2015	26361076	Bangladesh	Dhaka	Asia	Southern Asia	Hospital-based	1-Feb-2009	31-Mar-2013
Shahunja	2020	33264364	Bangladesh	Dhaka	Asia	Southern Asia	Hospital-based	1-Jun-2014	31-May-2017
Shimoni	1999	10825045	Israel	Petach Tikva	Asia	Western Asia	Hospital-based	1-Jan-1987	31-Dec-1996
Shkalim	2012	22161258	Israel	Tikva	Asia	Western Asia	Hospital-based	1-Nov-1995	31-Oct-2010
Sigauque	2009	19131902	Mozambique	Manhiça district	Africa	Eastern Africa	Hospital-based	1-May-2001	30-Apr-2006
Sirinavin	1999	10524956	Thailand	Bangkok	Asia	South-eastern Asia	Hospital-based	1-Jan-1978	31-Dec-1994
Still	2020	32725229	Mali	Bamako	Africa	Western Africa	Hospital-based and outpatient	1-Jun-2002	31-Dec-2018
Tabu	2012	22363591	Kenya	Asembo (rural)	Africa	Eastern Africa	Hospital-based	1-Oct-2006	30-Sep-2009
Tack	2020	32240161	Democratic Reput	Kisantu	Africa	Middle Africa	Hospital-based	1-Jan-2015	31-Oct-2017
Thamlikitkul	1996	8765451	Thailand	Bangkok	Asia	South-eastern Asia	Hospital-based	1-Jan-1993	31-Dec-1993
Tsai	2007	17901796	Taiwan	Taoyuan	Asia	Eastern Asia	Hospital-based	1-May-1996	30-Jun-2003
Vaagland	2004	15367335	Tanzania	Mbulu	Africa	Eastern Africa	Hospital-based	1-Jul-2000	31-Aug-2000
Vandenberg	2010	20104200	Democratic Republic of the Congo	Kivu Province	Africa	Middle Africa	Hospital-based	1-Jan-2002	31-Dec-2006
Verani	2015	26449945	Kenya	Lwak (rural)	Africa	Eastern Africa	Population-based	1-Jan-2009	31-Dec-2014

Vlieghe	2012	23272255	Cambodia	Phnom Penh	Asia	South-eastern Asia	Hospital-based	1-Jul-2007	30-Jun-2011
Walsh	2000	10783021	Malawi	Blantyre	Africa	Eastern Africa	Hospital-based	1-Sep-1996	31-Aug-1997
Wang	2006	16569283	Taiwan	Taipei	Asia	Eastern Asia	Hospital-based	1-Jan-1996	31-Dec-2004
Yen	2007	17639163	Taiwan	Taipei	Asia	Eastern Asia	Hospital-based	1-Jan-2001	31-Dec-2003
Yen	2009	19686996	Taiwan	Taipei	Asia	Eastern Asia	Hospital-based	1-Jan-2004	31-Dec-2006
Yombi	2015	25819116	Belgium	Brussels	Europe	Western Europe	Hospital-based	1-Jan-2007	31-Dec-2012
Zaidi	1999	10608627	United States	Boston, MA	Americas	Northern America	Hospital-based	1-Jan-1979	31-Dec-1995

*UN sub-regions with no data available include: Northern Africa, Caribbean, Central America, South America, Central Asia, Australia and New Zealand, Melanesia, Micronesia, Polynesia

Supplementary Table S2. Prevalence of non-typhoidal *Salmonella* serovars, global, 1971 through 2019

Serovar	Cases	Proportion of iNTS disease, %
Typhimurium	5,934	45·7
Enteritidis	4,112	31·7
Dublin	403	3·1
Virchow	186	1·4
Isangi	172	1·3
Choleraesuis	134	1·0
Heidelberg	90	0·7
Java	54	0·4
Oranienburg	47	0·4
Infantis	46	0·4
Newport	42	0·3
Stanley	38	0·3
Panama	36	0·3
Poona	36	0·3
Corvallis	27	0·2
Saintpaul	25	0·2
Schwarzengrund	22	0·2
Montevideo	21	0·2
Chester	19	0·1
Bovis-morbificans	17	0·1
Colindale	15	0·1
Krefeld	15	0·1
Brandenburg	15	0·1
Hadar	14	0·1
Agona	10	0·1
Copenhagen	5	<0·1
Berta	4	<0·1
Derby	3	<0·1
Senftenberg	3	<0·1
Abony	3	<0·1
Livingston	3	<0·1
Bredeney	3	<0·1
Minnesota	3	<0·1
Kentucky	3	<0·1
Irumu	2	<0·1
Urbana	2	<0·1
Bareilly	2	<0·1
Kedougou	2	<0·1

Sandiego	2	<0·1
Kisangani	2	<0·1
Anatum	2	<0·1
Braenderup	2	<0·1
Albany	2	<0·1
Ohio	2	<0·1
Stanleyville	1	<0·1
Coeln	1	<0·1
Uganda	1	<0·1
Give	1	<0·1
London	1	<0·1
Muenster	1	<0·1
Telelkibir	1	<0·1
Brancaster	1	<0·1
Vitiki	1	<0·1
Umbilo	1	<0·1
Senegal	1	<0·1
Kottbus	1	<0·1
Chandans	1	<0·1
Amsterdam	1	<0·1
Telaviv	1	<0·1
Hvittingfoss	1	<0·1
Mbandaka	1	<0·1
Reading	1	<0·1
Freetown	1	<0·1
Blockley	1	<0·1
Bardo	1	<0·1
Haifa	1	<0·1
Bertha	1	<0·1
Kibusi	1	<0·1
Javiana	1	<0·1
Other *Salmonella*^	1,372	10·6
**Total**	**12,977**	**100·0**

iNTS = Non-typhoidal *Salmonella* invasive disease
^ Non-typhoidal *Salmonella* serotyping performed but could not identify serovars or authors provided only top serovars and did not describe all serovars that were serotyped

Figure S1. Case fatality ratio by year in Manhiça, Mozambique, Africa, from 2001 through 2014 (Mandomando, et al. Clin Infect Dis 2015. DOI 10.1093/cid/civ712)

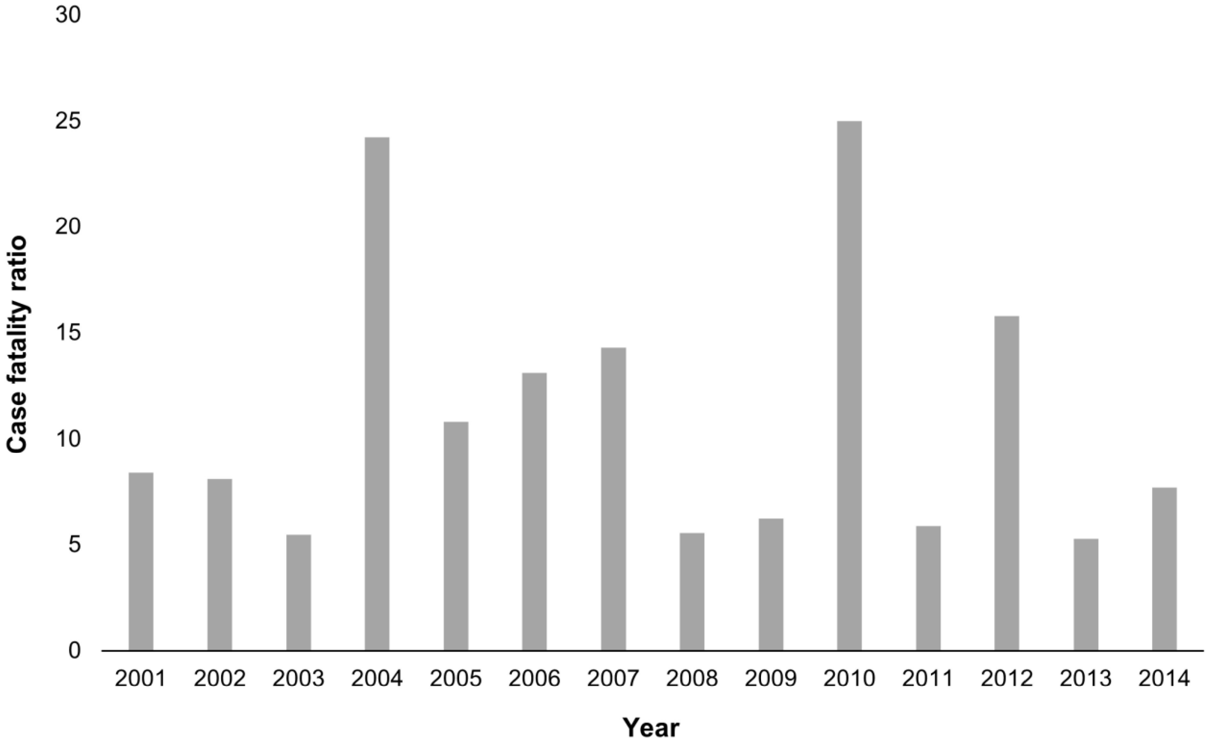

Figure S2A. Forest plot of the association between the risk of death among iNTS participants with and without HIV-coinfection

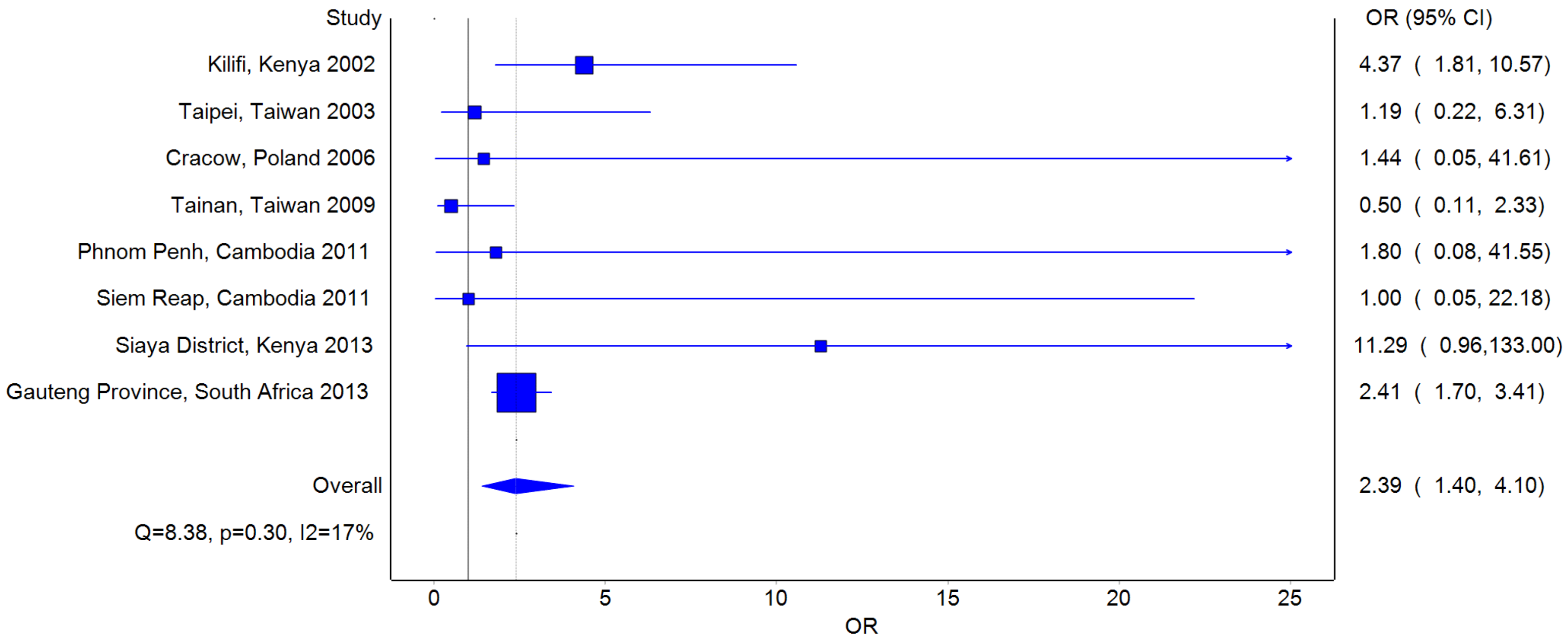

Figure S2B. Forest plot of the association between the risk of death among iNTS participants with and without malaria-coinfection

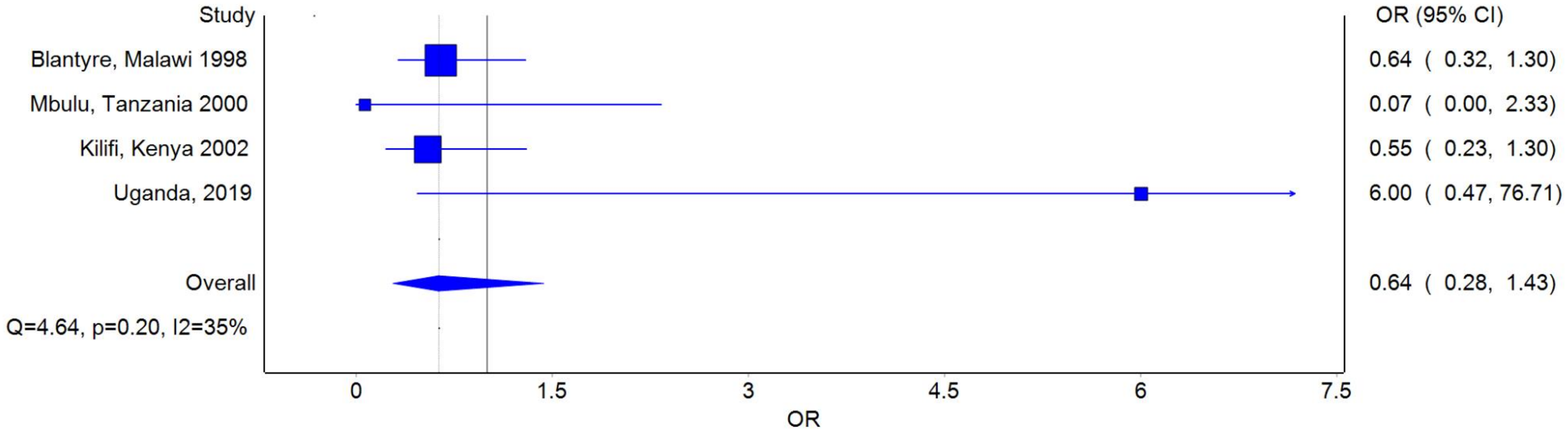

Supplement: Supplementary appendix [file mmc1.pdf]
